# A spiro-type self-assembled hole transporting monolayer for highly efficient and stable inverted perovskite solar cells and modules†

**DOI:** 10.1039/d4ee01960a

**Published:** 2024-11-27

**Authors:** Xianfu Zhang, Botong Li, Shaochen Zhang, Zedong Lin, Mingyuan Han, Xuepeng Liu, Jianlin Chen, Weilun Du, Ghadari Rahim, Ying Zhou, Pengju Shi, Rui Wang, Pengfei Wu, Thamraa Alshahrani, Wadha Alqahtani, Norah Alahmad, Qian Wang, Bin Ding, Songyuan Dai, Mohammad Khaja Nazeeruddin, Yong Ding

**Affiliations:** a Beijing Key Laboratory of Novel Thin-Film Solar Cells, North China Electric Power University Beijing 102206 China liuxuepeng@ncepu.edu.cn dingy@ncepu.edu.cn; b Institut des Sciences et Ingénierie Chimiques, Ecole Polytechnique Fédérale de Lausanne (EPFL) Lausanne CH-1015 Switzerland bin.ding@epfl.ch mdkhaja.nazeeruddin@epfl.ch; c School of Integrated Circuits, Southeast University Wuxi 214026 Jiangsu P. R. China; d School of Engineering, Westlake University Hangzhou 310024 China; e Computational Chemistry Laboratory, Department of Organic and Biochemistry, Faculty of Chemistry, University of Tabriz Tabriz 5166616471 Iran; f Department of Physics, College of Science, Princess Nourah bint Abdulrahman University Riyadh 11671 Saudi Arabia; g School of Materials Science and Engineering, Taizhou University Taizhou 318000 P. R. China; h State Key Laboratory for Strength and Vibration of Mechanical Structures, Xi’an Jiaotong University 710049 Xi’an P. R. China; i Institute for Advanced Materials and Technology, University of Science and Technology Beijing Beijing 100083 China b2286713@ustb.edu.cn

## Abstract

Self-assembled monolayers (SAMs) have significantly contributed to the advancement of hole transporting materials (HTMs) for inverted perovskite solar cells (PSCs). However, uneven distribution of SAMs on the substrate largely decreases the PSC performance, especially for large-scale devices. Herein, the first spiro-type SAM, termed 4PA-spiro, with an orthogonal spiro[acridine-9,9′-fluorene] as the skeleton and phosphonic acid as the anchoring group were proposed. Compared to the reference 4PACz, the twisted configuration with larger steric hindrance of 4PA-spiro inhibited the intermolecular aggregation, enabling a uniform and homogeneous anchoring on the substrate. Moreover, the suitable highest occupied molecular orbital (HOMO) level of 4PA-spiro is beneficial in promoting hole extraction and reducing charge non-radiative recombination. As a result, compared to 4PACz with a power conversion efficiency (PCE) of 22.10%, the 4PA-spiro-based PSCs exhibited a superior PCE of 25.28% (certified 24.81%, 0.05 cm^2^), along with excellent long-term stability. More importantly, 4PA-spiro-enabled larger-area PSCs and modules achieved PCEs of 24.11% (1.0 cm^2^) and 21.89% (29.0 cm^2^), respectively, one of the highest PCEs for inverted PSC modules, providing an effective SAM candidate for the commercialization of efficient, stable and large-scale inverted PSCs.

## Abstract 2

The twisted spiro-type SAM 4PA-spiro effectively suppresses molecular aggregation and ensures appropriate energy levels, providing an efficiency of 25.28 and 21.87% for the p–i–n PSCs (0.05 cm^2^) and modules (29.0 cm^2^).

## Graphical abstract

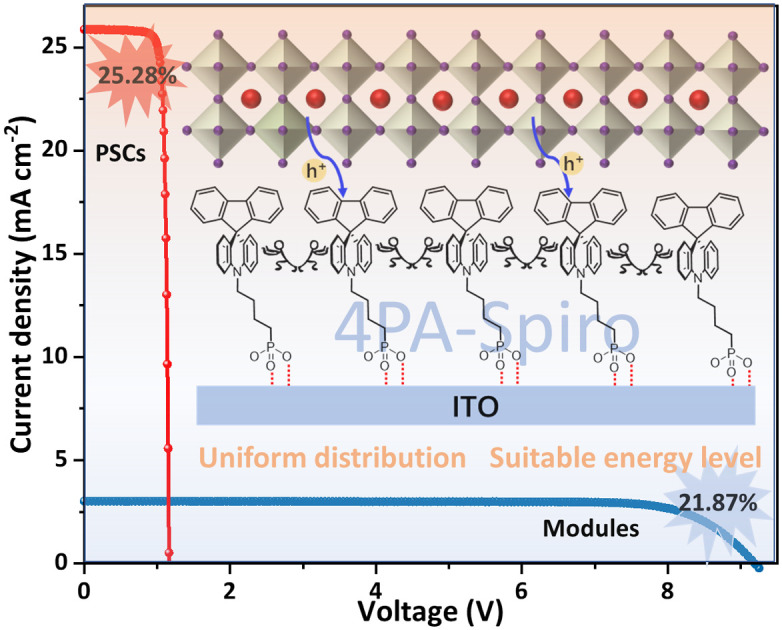

**Broader context**

Perovskite solar cells (PSCs) have emerged as a promising photovoltaic technology due to their high power conversion efficiency and relatively low fabrication costs. However, further enhancing their performance and stability is essential for widespread adoption. Efficient hole-transporting layers play a critical role in achieving these objectives. The development of 4PA-spiro self-assembled monolayers (SAMs) marks a significant advancement in the PSC field. Compared to conventional alternatives like 4PACz, 4PA-spiro-based PSCs demonstrate superior performance and achieve a power conversion efficiency (PCE) of 25.28%. Moreover, 4PA-spiro-based PSCs exhibit excellent long-term stability, which is crucial for their practical application and commercialisation.

## Introduction

Perovskite solar cells (PSCs) have attracted global attention due to their rapid increase in power conversion efficiency (PCE) and facile device fabrication.^1,2^ Recently, p–i–n structure single-junction and tandem PSCs (with silicon, CIGS, organic and perovskite) have gained significant progress, owing largely to the successful application of self-assembled monolayers (SAMs) as hole-transporting materials (HTMs).^3–9^ The SAMs offer various advantages, such as minimal electric and optical loss, easy modulation of energy levels, conformal coating on the substrate and simple film fabrication. The SAM molecule structure primarily comprises a skeleton, a spacer group, and an anchoring group.^10,11^ To date, due to the robust interaction of the phosphonate group with metal oxides, numerous molecular skeletons featuring phosphonic acid as an anchoring group have been successfully applied in SAMs, including carbazole and its analogues,^12–15^ dimethylacridine,^16^ and phenothiazine–triphenylamine,^17^ among others.^18,19^

SAMs employed in PSCs share a structural resemblance to the molecular dyes utilized in dye-sensitized solar cells (DSSCs).^10,20,21^ Early studies on DSSCs demonstrated that dye aggregation may substantially degrade device performance, especially dye clustering, which has the potential to disrupt the monolayer structure and energy levels.^22–25^ Moreover, a dye molecule anchored to the substrate surface (TiO_2_ in DSSC) is too close to another dye on the surface, causing molecule interactions.^22–25^ Similarly, SAMs, especially those based on planar carbazole molecules in PSCs, face challenges associated with excessive molecular aggregation, both in solution and film states, resulting in non-uniform SAM coating on substrates.^12,22,26–28^

Similar to the strategy used in DSSCs, where co-adsorbents are employed to mitigate dye aggregation on titanium oxides,^29,30^ Sargent *et al.* introduced 3-mercaptopropionic acid (3-MPA) into 2PACz to form co-adsorption, which decreases the number of SAM clusters and homogenizes the distribution of SAMs on the substrate.^31^ Planar dyes are inherently susceptible to aggregation in terms of the molecular structure of organic compounds, while twisted structures are advantageous for suppressing undesirable dye aggregation.^22,32–34^ Due to the steric repulsive interaction between the terminal aromatic rings, incorporating a partial distortion to carbazole-based SAMs leads to a non-coplanar screw-shaped configuration, which effectively hinders molecular aggregation.^12^ Moreover, extending the conjugated system for 2PACz by introducing more benzene units to form a twist structure, termed Ph-2PACz, leads to large intermolecular distances and decreased aggregation. The monolithically integrated perovskite-Si tandem solar cell using Ph-2PACz exhibits a PCE of 28.9% with superior stability.^35^ Besides, Ph-4PACz also exhibits similar results in single junction PSCs.^36^ In addition, introducing methyl-containing acridine as the molecular skeleton could also prevent aggregation and obtain a PCE of 25.86% (certified: 25.39%).^16^ On the other hand, in n–i–p PSCs, using 2,2′,7,7′-tetrakis[*N*,*N*-di(4-methoxyphenyl)amino]-9,9′-spirobifluorene (spiro-OMeTAD) retained the highest reported PCE due to the unique properties of the 3D spiro-bifluorene on the molecular center.^37^ The spiro structure has isotropic charge transfer characteristics, inhibited molecular aggregation, and easy formation of excellent ohmic contact with perovskite films.^38–41^

**Fig. 1:**
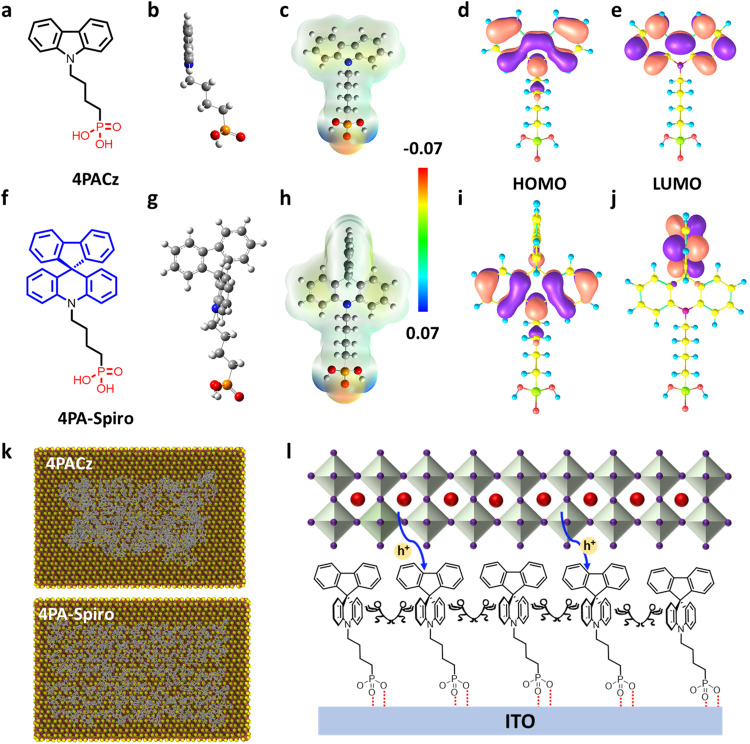
Chemical structure of (a) 4PACz and (f) 4PA-spiro. Optimized structure (side view) of (b) 4PACz and (g) 4PA-spiro. Electrostatic surface potentials of (c) 4PACz and (h) 4PA-spiro. Calculated HOMO orbital distributions of (d) 4PACz and (i) 4PA-spiro. Calculated LUMO orbital distributions of (e) 4PACz and (j) 4PA-spiro. (k) Top view of the equilibrated molecular representations of 4PACz and 4PA-spiro on the surface of In_2_O_3_. (l) Schematic illustration of 4PA-spiro as the HTM interacting with ITO and perovskite.

Thus, inspired by the orthogonal structure of classical spiro-OMeTAD, we designed a novel spiro-type SAM, named 4PA-spiro (Fig. 1f), featuring twisted spiro[acridine-9,9′-fluorene] as the skeleton group and phosphoric acid as the anchoring group. In comparison to 4PACz (Fig. 1a), the 3D-spiro structure of 4PA-spiro is more effective in suppressing molecular aggregation, resulting in a more uniform and homogeneous anchoring on the substrate. In addition, the suitable energy levels of 4PA-spiro facilitate charge carrier transport and suppress non-radiative recombination when compared to 4PACz. Employing the 4PA-spiro as the HTM in p–i–n PSCs yielded an impressive PCE of 25.28%, and superior long-term environmental stability. Besides, 4PA-spiro-enabled larger-area PSCs and modules achieved PCEs of 24.11% (1.0 cm^2^) and 21.89% (29.0 cm^2^), respectively, one of the highest PCEs for inverted PSC modules.

## Results and discussion

The synthesis route for 4PA-spiro is similar to recently reported SAMs in organic solar cells and PSCs.^12,13,42^ Scheme S1 (ESI†) shows detailed synthesis routes, and the target 4PA-spiro delivers a high overall yield of 63% from the initial raw material (from 10*H*-spiro[acridine-9,9′-fluorene]), and the estimated synthesis cost is just around 6.53 $ per g. The molecular structure is characterized by NMR (^1^H, ^13^C, and ^31^P) spectra and HRMS (Fig. S1–S5, ESI†). The decomposition temperature of 4PA-spiro exceeds 270 °C (Fig. S6, ESI†), illustrating its high thermal stability. In addition, compared to the glass transition temperature (*T*_g_) of 81 °C for 4PACz (Fig. S7, ESI†), the higher *T*_g_ at 141 °C for 4PA-spiro promotes the formation of a stable amorphous film and increases film coverage on surfaces.^43^ Using SAMs as the window layer of the devices, we investigated the UV-vis absorption and ^1^H NMR spectra of the molecules before and after illumination, confirming that the novel 4PA-spiro exhibits illumination stability (Fig. S8 and S9, ESI†).

As illustrated in Fig. 1f, 4PA-spiro retains the spiro structure (blue) while combining it with the phosphoric acid anchoring group (red) in the conventional SAM configuration. The optimized structure, as depicted in the side view (Fig. 1b and g) and top view (Fig. S10, ESI†), further illustrates the orthogonal configuration of 4PA-spiro. To gain further insight into its intermolecular π–π interactions, X-ray single crystals of spirobifluorene (CCDC 273066) and 9*H*-carbazole (CCDC 10242020) were analyzed. The twisted spiro-bifluorene exhibits a 3D spatial structure with significantly longer intermolecular distances (Fig. S11, ESI†), which would effectively suppress molecular aggregation. In addition, as shown in Fig. S12 (ESI†), the improved solubility of 4PA-spiro demonstrates that the twisted molecular backbone may inhibit self-aggregation of the unfused-ring electron acceptor.^12^

Additionally, molecular dynamics simulation was performed to investigate the adsorption process of SAM molecules on the ITO substrate.^44^ As illustrated in Fig. 1k, from the top view of the equilibrated molecule representations on the surface of In_2_O_3_, 4PA-spiro exhibits less molecular aggregation than 4PACz, resulting in higher surface coverage. Moreover, Fig. S13 (ESI†) indicates that during the first 1.3 nanoseconds of the simulation, the final numbers of clusters for 4PACz and 4PA-spiro have reached 95% and 87%, respectively, confirming that the spiro-structure is beneficial to inhibit the formation of clusters. A schematic diagram was proposed to understand the molecular characteristics of 4PA-spiro (Fig. 1l). The 4PA-spiro is expected to form a homogeneous film on the ITO substrate, attributed to the ideal steric hindrance of the terminal spiro[acridine-9,9′-fluorene]. This hindrance is expected to impede molecular aggregation.

Electrostatic surface potentials (ESPs) show that 4PA-spiro exhibits a charge density comparable to 4PACz (Fig. 1c and h). However, the fluorene region, distant from the phosphoric acid, has a slightly different electron density. As confirmed by electron distribution (Fig. 1d, e, i and j), the highest occupied molecular orbital (HOMO) and lowest unoccupied molecular orbital (LUMO) of 4PACz are mostly concentrated on the carbazole unit. Interestingly, the HOMO of 4PA-spiro is localized on the acridine unit, but the LUMO is mainly concentrated on the fluorene unit, enabling the fast formation of neutral excitation and hole transfer transitions in 4PA-spiro.^45,46^

Calculated HOMO/LUMO energy levels from time-dependent density functional theory (TDDFT) calculation of 4PACz are −5.59 eV/−0.98 eV, respectively. Encouragingly, 4PA-spiro exhibits high energy levels, with calculated HOMO/LUMO energy levels of −5.25 eV/−1.07 eV, respectively. Therefore, 4PA-spiro may have more suitable energy level alignment with the perovskite layer, which is almost identical with prior studies of carbazole molecular tailoring on SAMs.^13,35^

**Fig. 2:**
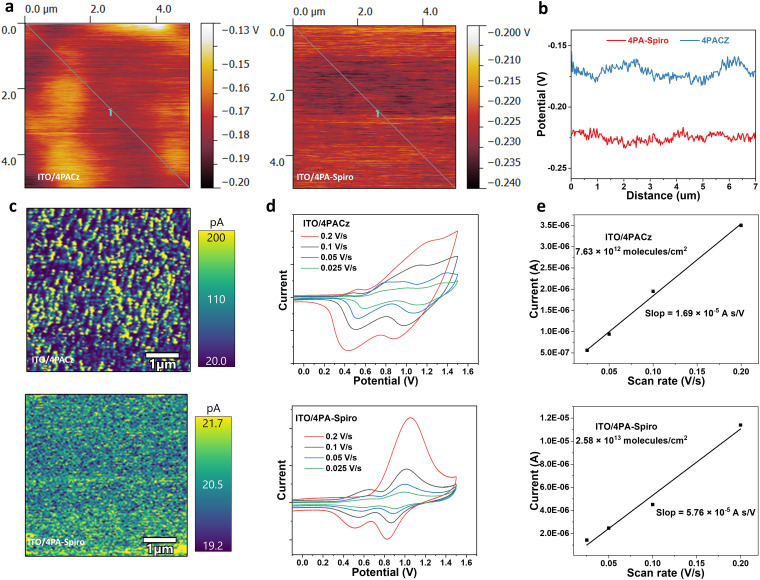
(a) Kelvin probe force microscopy (KPFM) images and (b) curves of the ITO anchoring with different SAMs. (c) C-AFM of 4PACz and 4PA-spiro coated on the ITO substrate. (d) CV curves of SAMs with different scan rates. (e) Molecular adsorption density calculation by CV measurements.

The morphology of the SAMs on the substrate was confirmed using atomic force microscope (AFM) measurements (Fig. S14, ESI†). Upon deposition of 4PA-spiro on the ITO substrate, the root mean square (RMS) value of the substrate decreased from 2.57 nm to 1.36 nm, whereas 4PACz had a higher RMS of 1.78 nm, indicating that steric hindrance from 4PA-spiro results in a more uniform and homogeneous film on ITO than 4PACz.^12,47^ Besides, because of the monolayers of the HTMs, the ITO substrate with different SAMs exhibited similar roughness after rinsing the unbonded molecules. Kelvin probe force microscopy (KPFM) was employed to investigate the surface potential with contact potential differences (CPD). Fig. 2a and b demonstrate that 4PA-spiro exhibits a more uniform potential distribution than 4PACz. Besides, Conductive atomic force microscopy (C-AFM) measurements were further conducted for the SAM-coated ITO substrates. Fig. 2c shows that 4PA-spiro exhibits slightly lower conductivity than 4PACz, possibly due to reduced molecule interaction. However, because of the ultra-thin thickness of the SAMs, it has a minimal impact on charge transfer.^11^ Moreover, the color contrast in Fig. 2c shows that 4PA-spiro has a narrower current distribution than 4PACz, demonstrating that 4PA-spiro is conducive to suppressing molecular aggregation and promoting uniform distribution on the ITO substrate. To evaluate the surface density of SAM molecules on ITO, cyclic voltammetry (CV) measurements with different scan rates were conducted.^14^ As shown in Fig. 2d and e, compared to 7.63 × 10^12^ molecules per cm^2^ for 4PACz, 4PA-spiro presents a higher molecular packing density of 2.58 × 10^13^ molecule per cm^2^. This indicates that the large steric spiro structure promotes molecule adsorption on the substrate.

To investigate perovskite film growth on the different SAM-coated ITO substrates, scanning electron microscopy (SEM) measurements were conducted on the corresponding ITO/SAM/perovskite films. Fig. S15 and S16 (ESI†) show the buried and top surfaces of the perovskite films. Due to the excellent wetting performance of the perovskite precursor solution on the SAM coated substrate, both SAMs exhibit similar smooth buried morphology, which is advantageous for hole collection and transfer at the SAM/perovskite interface. Moreover, cross-sectional SEM images of 4PACz and 4PA-spiro-based PSCs (Fig. S17, ESI†) display that the perovskite is in close contact with a tight, non-porous interface, consistent with the buried interface. Simultaneously, the SAMs have a very low thickness, as previously described due to the formation of the monolayer.^31,48^ This is advantageous in reducing the impact of the HTM on the spectrum absorption of the perovskite. The heights of optimal molecular structures on the substrate with different stacking arrangements were determined using DFT calculation, and the thickness of 4PA-spiro ranges from 0.99 to 1.58 nm (Fig. S18, ESI†). In addition, as evidenced by AFM measurements, the thickness of the SAM layer deposited on a silicon wafer was ∼2 nm, which is consistent with the findings from DFT calculation (Fig. S19, ESI†).^49^

The wetting performance of the perovskite precursor solution on the SAM-coated ITO substrate and the SAMs’ hydrophobicity were evaluated using contact angle measurements. The contact angles of the perovskite precursor solution on the 4PACz and 4PA-spiro surface were determined to be 32° and 29°, respectively (Fig. S20, ESI†), indicating that the novel 4PA-spiro is suitable as a SAM for the perovskite film fabrication.^50^ Furthermore, 4PA-spiro and 4PACz exhibit similar hydrophobic properties (Fig. S21, ESI†), with water contact angles of around 80°, which is beneficial for improving the humidity stability of the devices.^13,46^

**Fig. 3:**
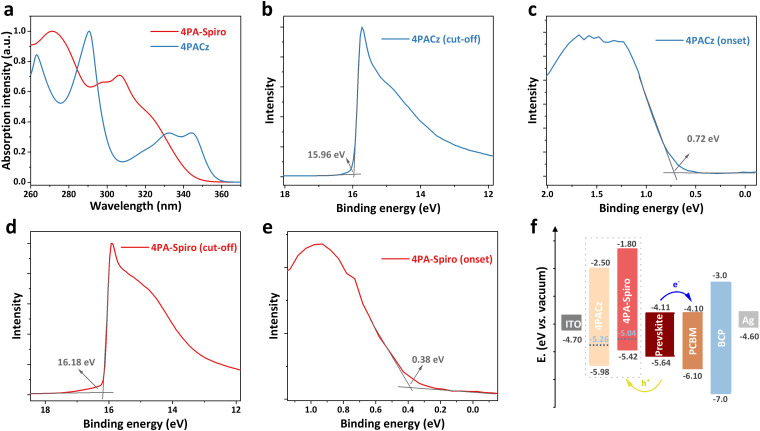
(a) UV-vis absorption spectra of 4PA-spiro and 4PACz in DMF solvent. (b) Cut-off and (c) onset region of the UPS spectra for 4PACz. (d) Cut-off and (e) onset region of the UPS spectra for 4PA-spiro. (f) Energy level diagram of PSCs with different SAMs.

**Fig. 4:**
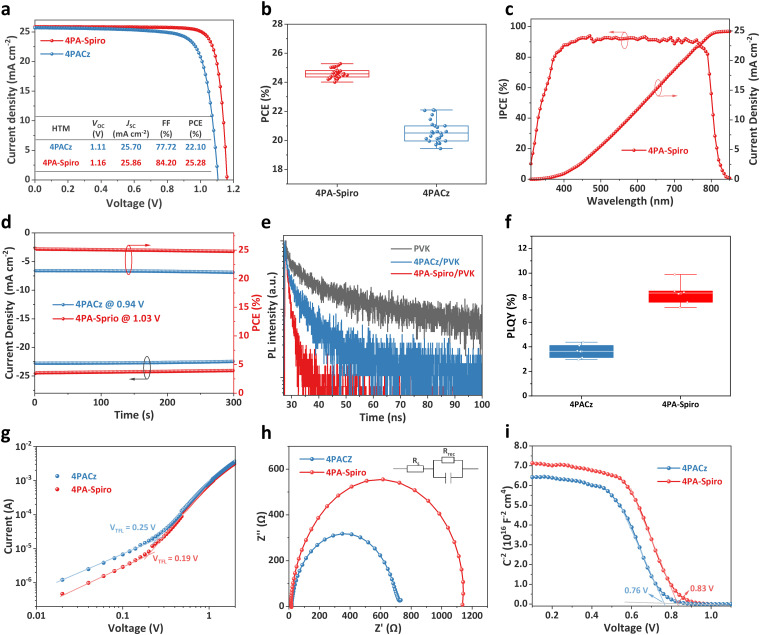
(a) *J*–*V* curves of the best-performing PSCs with 4PACz and 4PA-spiro. (b) Statistical distribution of PCEs for 4PACz and 4PA-spiro based PSCs. (c) IPCE spectra and integrated *J*_SC_ curve of 4PA-spiro based PSCs. (d) Continuous maximum-power point tracking for encapsulated PSCs under AM 1.5 G illumination in ambient air. (e) TRPL decays of the perovskite on SAMs at the buried interface. (f) PLQY of the perovskite films deposited on different SAMs. (g) SCLC, (h) EIS and (i) Mott–Schottky plots of PSCs with 4PACz and 4PA-spiro as the HTMs.

To investigate the energy levels of the SAMs, UV-vis absorption and ultraviolet photoelectron spectroscopy (UPS) measurements were conducted. As shown in Fig. 3a, the UV-vis absorption edges of 4PA-spiro and 4PACz are located at 343 and 356 nm, respectively. The corresponding energy gaps (*E*_g_) are calculated to be 3.62 and 3.48 eV for 4PA-spiro and 4PACz, respectively. Meanwhile, through the cut-off region of the UPS spectra for 4PACz (Fig. 3b) and 4PA-spiro (Fig. 3d), the corresponding Fermi levels are determined to be −5.26 and −5.04 eV, respectively. And the HOMO levels are determined to be −5.42 and −5.98 eV for 4PA-spiro and 4PACz, respectively, based on the onset value of 0.72 and 0.38 eV in Fig. 3c and e. Based on LUMO = *E*_g_ + HOMO, the LUMO levels of 4PACz and 4PA-spiro are −2.50 and −1.80 eV respectively, which efficiently blocks the electron and inhibits charge nonradiative recombination. The valence band maximum (VBM) and conduction band minimum (CBM) of the perovskite were determined to be −5.64 and −4.11 eV, respectively (Fig. S22, ESI†). The corresponding energy level diagram is thus shown in Fig. 3f. In comparison to the valence band of the perovskite, the HOMO level of 4PA-spiro is more suitable for hole carrier transfer than 4PACz, which has the potential to provide higher open-circuit voltage (*V*_OC_) for PSCs.^51^

The performance of the SAMs in PSCs was investigated using the ITO/SAMs/Cs_0.05_MA_0.05_FA_0.9_PbI_3_/PCBM/BCP/Ag structure. Fig. S23 (ESI†) shows the optimal SAM concentrations of PSCs, which are 1.0 mg mL^−1^ for 4PA-spiro and 0.5 mg mL^−1^ for 4PACz. To further investigate the potential of SAMs in PSCs, top surface passivation and an anti-reflective film were used on both 4PA-spiro and 4PACz based PSCs.^16,52^ As shown in Fig. 4a, 4PA-spiro based devices have a higher champion PCE of 25.28% than 4PACz based devices (PCE = 22.10%). The PCE distribution delivers an average efficiency of 24.64% for 4PA-spiro-based devices, higher than that of 20.61% for 4PACz-based devices (Fig. 4b and Fig. S24, ESI†). Moreover, PCEs of 4PA-spiro-based devices have a narrower distribution range than those of 4PACz, indicating that 4PA-spiro promotes uniform coverage and improves reproducibility. The hysteresis behaviors of 4PACz and 4PA-spiro-based devices with different scan directions were studied (Fig. S25, ESI†). The 4PA-spiro-based devices exhibited a negligible hysteresis index (HI) of 0.5% compared to 4.2% of the 4PACz-based devices. To ensure the reliability of the findings, 4PA-spiro-based devices were sent to a third-party certification laboratory. Fig. S26 (ESI†) shows that an average certified PCE of 24.81% was achieved, along with a quasi-steady-state efficiency of 24.61%, which is consistent with the before mentioned *J*–*V* measurements.

In addition, as shown in Fig. S27 (ESI†), the typical SAMs reported for high-efficiency PSCs are also investigated, including 2PACz,^53^ Me-4PACz,^54^ MeO-2PACz,^55^ 4PADCB,^12^ Ph-2PACz,^35^ Ph-4PACz^56^ and DMAcPA.^16^ Contrasted with the PCE exceeding 25% for 4PA-spiro-based PSCs, Fig. S28 (ESI†) demonstrates lower PCEs of 23.73, 23.22, 23.49, 23.86, 23.85, 24.04, and 24.42% obtained for 2PACz, Me-4PACz, MeO-2PACz, 4PADCB, Ph-2PACz, Ph-4PACz, and DMAcPA-based devices, respectively. This further underscores the significant potential of 4PA-spiro as SAMs for PSCs.

Incident-photon-to-current efficiency (IPCE) measurement was conducted for the PSCs with different SAMs (Fig. 4c and Fig. S29, ESI†). The integrated current density of the 4PACz and 4PA-spiro-based devices was determined to be 24.60 and 24.91 mA cm^−2^, respectively, consistent with the short-circuit current density (*J*_SC_) obtained from the *J*–*V* measurement. Steady-state PCE was performed further to verify operational stability under AM 1.5 G illumination. Fig. 4d displays that the 4PA-spiro and 4PACz-based devices achieve stabilized efficiencies of 24.77% and 21.11%, respectively, over 300 seconds, indicating that both SAMs exhibit high light soaking stability.

To investigate the dynamics of charge transfer at the perovskite/HTM interface, steady-state photoluminescence (PL) measurements were performed on ITO/SAMs/perovskite films at the bottom interface from the ITO side. 4PA-spiro has a higher fluorescence quenching efficiency of 84.2%, compared to 57.5% for 4PACz (Fig. S30, ESI†). Besides, the time-resolved PL (TRPL) spectra reveal that the lifetimes of the bare perovskite, 4PACz/perovskite, and 4PA-spiro/perovskite films are 11.7, 5.7, and 2.1 ns, respectively (Fig. 4f). The higher quenching efficiency and shorter lifetime of the 4PA-spiro/perovskite film indicate faster hole transfer and suppressed charge recombination at the perovskite/4PA-spiro interface compared to the 4PACz/perovskite film.^28^ In addition, Fig. 4f shows that the 4PA-spiro-based perovskite film has a higher value of photoluminescence quantum yield (PLQY) than 4PACz, confirming the low *V*_OC_ loss at the buried interface of 4PA-spiro.^44,57^

Light intensity-dependent *V*_OC_ measurements were carried out to study the charge recombination process in the 4PACz and 4PA-spiro-based devices. The 4PA-spiro-based devices have a lower ideal factor (*n*) of 1.34 than that of 1.67 for the 4PACz based devices, indicating less charge non-radiative recombination at the 4PA-spiro/perovskite interface (Fig. S31, ESI†). Furthermore, the space-charge-limited-current (SCLC) measurement was performed using an ITO/SAMs/perovskite/spiro-OMeTAD/Au structure to investigate trap densities. As shown in Fig. 4g, the trap filled limited voltage (*V*_TFL_) of the 4PACz and 4PA-spiro-based devices are determined to be 0.25 and 0.19 V, respectively, and the corresponding trap densities (*N*_trap_) are estimated to be 4.91 × 10^−15^ and 2.01 × 10^−15^ cm^−3^ using the formula *N*_trap_ = 2*ε*_r_*ε*_0_*V*_TFL_/*qL*^2^. The reduced trap density in the 4PA-spiro-based devices might be attributed to the better coverage of 4PA-spiro on ITO, which inhibits direct contact between ITO and the perovskite.

The Nyquist plots of PSCs with different HTMs were analyzed from electrochemical impedance spectra (EIS) (Fig. 4h). The 4PA-spiro and 4PACz-based devices have comparable series resistance (*R*_s_) of 17.4 Ω and 20.2 Ω, respectively. However, the 4PA-spiro-based device has a much higher recombination resistance (*R*_rec_) of 1086 Ω compared to the 4PACz-based device (669 Ω). This increase in *R*_rec_ suggests more efficient charge extraction and less charge recombination in the 4PA-spiro-based devices. Moreover, Fig. 4i shows the Mott–Schottky plots of the 4PA-spiro and 4PACz based devices, and the 4PA-spiro-based device has a higher built-in potential (*V*_bi_) of 0.83 V than that of 0.76 V for the 4PACz-based device. This higher *V*_bi_ confirms that 4PA-spiro has a stronger driving force for carrier separation and transfer, which is consistent with the findings of the EIS measurements.

**Fig. 5:**
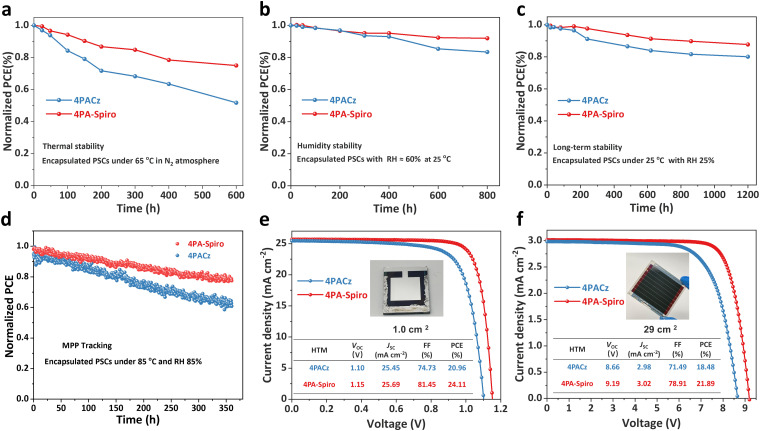
(a) Thermal stability (original PCEs of 24.76% for 4PA-spiro and 21.34% for 4PACz), (b) humidity stability (original PCEs of 25.12% for 4PA-spiro and 21.64% for 4PACz), (c) long-term environmental stability (original PCEs of 24.82% for 4PA-spiro and 22.03% for 4PACz) and (d) operational stability (original PCEs of 24.54% for 4PA-spiro and 20.72% for 4PACz) of the encapsulated 4PACz and 4PA-spiro-based devices. (e) *J*–*V* curves of large-area (1.0 cm^2^) PSCs. (f) *J*–*V* curves of PSC modules (29.0 cm^2^).

Stability is another critical aspect for practical PSC applications. Fig. 5a shows that the encapsulated 4PA-spiro-based device maintains 75% of its initial efficiency after 600 hours of storage at 65 °C in a nitrogen atmosphere, but the control 4PACz-based device only retains 52% of its initial efficiency. The improved thermal stability of the 4PA-spiro-based device is primarily attributed to its unique orthogonal rigid conformation, which elevates molecular thermal stability while decreasing interfacial defects.^58–60^Fig. 5b displays the humidity stability of the encapsulated 4PA-spiro and 4PACz-based devices. The 4PA-spiro-based device maintains 92% of its initial efficiency after 800 hours of operation at 60% relative humidity (RH) and 25 °C, while the 4PACz-based device maintains 82% of the initial efficiency. Furthermore, the long-term environmental stability of the 4PA-spiro and 4PACz-based devices was monitored at 25 °C and 25% RH. After 1200 hours, the 4PA-spiro-based device retains 88% of its initial efficiency, whereas the 4PACz-based device maintains 80% of its initial efficiency (Fig. 5c). The enhanced long-term environmental stability of the 4PA-spiro-based devices is attributed to the higher coverage and inhibition of SAM aggregation on the ITO substrate, resulting in decreased interface defects and improved device stability.^60^ The 4PA-spiro-based device maintains ∼80% of its original efficiency after 360 hours of continuous exposure to a white LED lamp (100 mW cm^−2^) at the maximum power point (MPP) tracking, under conditions of 85 °C and 85% RH (Fig. 5d). These findings demonstrate the development of a new class of SAMs based on spiro-type that are beneficial for p–i–n device stability, due to improved molecular thermal stability and SAM coverage on the ITO substrate.

Finally, the fabrication of large-scale PSCs is crucial for the commercialization of PSCs, and the 4PACz and 4PA-spiro are introduced into the large-area PSCs and PS modules with an aperture area of 1.0 and 29.0 cm^2^, respectively. As shown in Fig. 5e and Fig. S32 (ESI†), compared to 20.96% of 4PACz-based devices, 4PA-spiro based PSCs show a higher champion PCE of 24.11% (1.0 cm^2^) with *V*_OC_ of 1.15 V, *J*_SC_ of 25.69 mA cm^−2^ and FF of 81.45%. Besides, as exhibited in Fig. 5f and Fig. S33 (ESI†), a champion PCE of 21.89% for the 4PA-spiro-based PS modules is also acquired with *V*_OC_ of 9.19 V, *J*_SC_ of 3.02 mA cm^−2^ and FF of 78.91%, which is one of the highest PCEs for inverted PSC modules (Table S5, ESI†). The above results agree well with the *J*–*V* measurement for small area PSCs. The excellent PCEs of large-scale PSCs further illustrate the huge potential of 4PA-spiro as the HTM candidates for commercial inverted PSC applications.

## Conclusion

In summary, we effectively adopted a molecule design strategy that included a spiro structure to inhibit molecular aggregation, facilitating the uniform adsorption of SAMs on the substrate. Additionally, the energy levels of the novel SAMs, 4PA-spiro, are more closely matched with the perovskite, promoting efficient charge carrier transport while suppressing charge non-radiative recombination at the interface. Ultimately, the 4PA-spiro-based devices achieved an impressive efficiency of 25.28% (certified 24.81%) while maintaining excellent stability, indicating a substantial progress for efficient p–i–n PSCs. Moreover, the 4PA-spiro based large area PSCs and modules also shown excellent PCEs of 24.11 and 21.89%, respectively, showing a huge potential in the large-scale p–i–n PSCs.

## Author contributions

X. Z., B. L., S. Z. and Z. L. contributed equally to this work. X. L. designed and synthesized the molecules. X. Z. fabricated and tested efficient devices, and performed NMR measurement, CV measurement, and AFM measurements. B. L., J. C., W. D. and Y. Z. carried out the potential application of 4PA-spiro from over 10 materials in different batches of PSCs, S. Z., P. S. and R. W. tested and analyzed C-AFM and UPS tests. M. H. tested angle contact, transmittance, and absorption measurement. G. R. carried out the DFT calculation of molecular structure optimization, energy level and ESP. Z. L., P. W. and Q.W. collaboratively performed the molecular dynamics calculations of the adsorption process. Specifically, Z. L. constructed the adsorption substrate model and optimized the molecular structures; P. W. performed the calculations of the adsorption process; and Q. W. proposed the calculation strategy, wrote the corresponding section of the manuscript, and supervised the calculations. T. A., W. A. and N. A. conducted the SEM, EIS and Mott–Schottky measurements, X. Z. and B. D. fabricated and tested the large-scale PSCs, and B. L. conducted the stability measurements. S. D. and M. K. N. provided the financial support. Y. D. directed the fabrication of the efficient devices. X. Z., X. L., W. Q., B. L. and M. K. N. wrote the manuscript, and all authors contributed to the data analysis and commented on the manuscript. X. L., W. Q., B. D., Y. D. and M. K. N. conceived the research project and supervised different parts of the work.

## Data availability

The data are available and submitted as ESI.†

## Conflicts of interest

There are no conflicts to declare.

## Supplementary Material

EE-018-D4EE01960A-s001
