# Supplementary material for: A spiro-type self-assembled hole transporting monolayer for highly efficient and stable inverted perovskite solar cells and modules
Source: Energy Environ Sci. 2024 Nov 27;18(1):468–77. doi: 10.1039/d4ee01960a (PMC11619813; doi:10.1039/d4ee01960a)
Supplement: EE-018-D4EE01960A-s001 [file EE-018-D4EE01960A-s001.pdf]

Supplementary Information (SI) for Energy & Environmental Science.
This journal is © The Royal Society of Chemistry 2024

Supporting Information

Spiro-type Self-assembled Hole Transporting Monolayer for Highly Efficient and Stable Inverted Perovskite Solar Cells and Modules

Xianfu Zhang,[‡ab] Botong Li,[‡a] Shaochen Zhang,[‡d] Zedong Lin,[‡g] Mingyuan Han,[a] Xuepeng Liu,*[a] Jianlin Chen,[a] Weilun Du,[a] Ghadari Rahim,[e] Ying Zhou,[a] Pengju Shi,[d] Rui Wang,[d] Pengfei Wu,[h] Thamraa Alshahrani,[f] Wadha Alqahtani,[f] Norah Alahmad,[f] Qian Wang,*[i] Bin Ding,*[b] Songyuan Dai,[a] Mohammad Khaja Nazeeruddin*[bc] and Yong Ding*[ab]

[a] *Beijing Key Laboratory of Novel Thin-Film Solar Cells, North China Electric Power University, Beijing, 102206, China.*

Email: liuxuepeng@ncepu.edu.cn, dingy@ncepu.edu.cn

[b] *Institut des Sciences et Ingénierie Chimiques, Ecole Polytechnique Fédérale de Lausanne (EPFL), CH-1015 Lausanne, Switzerland.*

E-mail: bin.ding@epfl.ch, mdkhaja.nazeeruddin@epfl.ch

[c] *Chemistry Department, Faculty of Science, King Abdulaziz University, P. O. Box 80203, Jeddah, 21589, Saudi Arabia.*

[d] *School of Engineering, Westlake University, Hangzhou 310024, China.*

[e] *Computational Chemistry Laboratory, Department of Organic and Biochemistry, Faculty of Chemistry, University of Tabriz, Tabriz, 5166616471, Iran.*

T. Alshahrani, W. Alqahtani, N. Alahmad

[f] *Department of Physics, College of Science, Princess Nourah bint Abdulrahman University, Riyadh 11671, Saudi Arabia.*

[g] *School of Materials Science and Engineering, Taizhou University, Taizhou 318000, PR China.*

[h] *State Key Laboratory for Strength and Vibration of Mechanical Structures, Xi'an Jiaotong University, 710049, Xi'an, P.R. China.*

[i] *Institute for Advanced Materials and Technology, University of Science and Technology Beijing, Beijing 100083, China.*

Email: b2286713@ustb.edu.cn

[‡]*These authors contributed equally to this work.*

Experimental section

Materials

Unless explicitly stated, all chemicals, reagents, and solvents employed in synthesized materials and fabricating PSCs were obtained from commercial suppliers in regent-grade quality and utilized without additional purification. All reactions were conducted under a nitrogen atmosphere, employing standard Schlenk line techniques to ensure an inert and controlled environment. For the materials used for device fabrication, Lead (II) iodide (PbI_2) are purchased from TCI Corp. Formamidinium iodide (FAI), and Methylazanium iodide (MAI) are purchased from Greatcell Solar. The Cesium iodide (CsI), BCP, Methylazanium chloride (MACl)and PEABr are purchased from Xi'an Polymer Light Technology Corp, China. BCP is purchased from Advanced Election Technology CO., Ltd, China. PCBM is bought from Luminescence technology corp, China. All the dissolving and processing solvents including N, N-dimethylformamide (DMF), dimethyl sulfoxide (DMSO), chloroform, chlorobenzene (CB), isopropanol (IPA) are anhydrous and are purchased from Sigma-Aldrich, United States. For the materials used for preparing 4PA-Spiro, 10H-spiro[acridine-9,9'-fluorene] is purchased from Bidepharm, China. 1,4-dibromobutane, tetrabuthylammonium bromide, dichloromethane, triethyl phosphite, bromotrimethylsilane were bought from Innochem, China.

Device fabrication

The ITO (15 Ω) is cleaned with detergent, deionized water and ethanol for 15 minutes using the ultrasonic bath method. Before the deposition of SAMs, the substrate is processed with UV-Ozone for 10 minutes. The SAM film is prepared by spin-coating with 3000 r/m for 30 seconds in DMF solvent. After that, the film is heated at 100 °C for 10 minutes and washed with IPA. The perovskite precursor consisted of 1.6 M PbI_2, 1.4 M FAI, 0.07 M CsI, 0.07 M MAI, 0.2 M MACl, 1 mL DMSO and DMF solution with volume ratio of 8:2. The perovskite film is deposited by spin-coating with 1000 r/s for 10 seconds and 5000 r/s for 30 seconds. At the last 10 seconds, 150 μL of chlorobenzene solution was dropped on the perovskite. Then the film is annealed at 100

°C for 30 minutes. After cooled to room temperature, 1 mg mL^{-1} PEABr in IPA and DMF solvent (200:1) is deposited by spin-coating with 5000r/s and heated at 100 °C for 5 minutes.[1] The PCBM (20 mg mL^{-1} in CB) is prepared by spin-coating with 1500 r/s for 30 seconds. After that, the BCP (0.5 mg mL^{-1} in IPA) is deposited with 5000 r/s for 30 seconds. Finally, Ag electrode with thickness of 80 nm was prepared by vacuum evaporation. For the operational stability of MPP tracking under 85 °C and 85% RH, the C_{60} (30 nm) and atomic layer deposition SnO_2 (20 nm) structure were conducted to replace the PCBM and BCP as the electron transporting materials. Additionally, the Ag electrode was replaced with Au to prevent corrosion of the perovskite by the metal electrode. The preparation process of large-area PSCs is the same as that of small-area PSCs with aperture area of 1.0 cm^2.

For the PS modules including 8 sub-cells connected in series, the perovskite layer was prepared using a previously reported vacuum-assisted method.[2] Preparation of the electron transporting layers, HTMs and electrodes are the same as those used for small area cells. For laser etching in PS modules, the P1 lines were pre-patterned on the FTO subtract with a width of 60 μm using 60% laser power and a speed of 300 mm/s with a frequency of 65 kHz and a pulse width of 120 ns. The P2 lines were patterned before the Ag evaporation process with an average laser power of 5% at a speed of 1000 mm/s and a frequency of 100 kHz and a pulse duration of 120 ns. The P3 lines (with a width of 100 μm) were fabricated under the same scribing condition as the P2 lines after the deposition of the Ag layer.

Characterization

NMR spectra were recorded on a Brücker spectrometer (400 MHz). HRMS was performed on a Bruker Autoflex instrument. UV-vis spectra of investigated molecules are carried out on a UV-vis spectrophotometer (UV-3600 plus, Shimadzu Co. Ltd, Japan). The PL measurements of HTM were recorded on the fluorescence spectrophotometer (Hitachi F-4600, Japan). The PL spectrum of perovskite/HTM films were obtained from a fluorescence spectrometer (FLS980, Edinburgh, UK) with the excitation wavelength of 488 nm. The film morphology was measured by atomic force

microscopy (5500AFM, Agilent Technologies, USA). The *J–V* characteristics were carried out using a Keithley 2400 source meter under AM 1.5G and a 3A grade solar simulator (Newport, USA) with an intensity of 100 mW cm^{-2}. The incident photon-to-current conversion efficiency (IPCE) was recorded on QE/IPCE measurement kit (Sofn Instruments Co., LTD, China). The wetting property of HTMs or perovskite precursor was measured on a contact angle tester (METATEST E3300). The decomposition temperature of 4PA-Spiro is recorded on the TGA analyzer (NETZSCH TG 209F3). SEM images were obtained by using a scanning electron microscope (SU8010 Hitachi). Electrochemical impedance spectroscopy (EIS) and Mott-Schottky plots were performed using a two-electrode system under dark with Bio-Logic SP200 electrochemical workstation.

Molecular dynamics simulations

The molecular dynamics model was constructed and optimized using Materials Studio. 4PACz and 4PA-Spiro were randomly distributed on the surface of In_2O_3, with the model system dimensions being 127 Å × 82 Å × 33 Å. The two considered systems, 4PACz and 4PA-Spiro, consist of a total of 17,845 and 17,980 atoms, respectively. Classical molecular dynamics simulations with full atomic resolution were performed using the LAMMPS program.[3] The equations of motion were integrated using the velocity Verlet method with a time step of 1 fs.[4] To maintain isothermal conditions, the Nosé-Hoover thermostat was used with a time constant equal to 0.1 fs. Simulations were carried out in the NVT ensemble (constant number of particles, volume, and temperature) at a temperature of 300 K. Periodic boundary conditions were applied in the x and y directions, while reflective boundary conditions were used in the z direction. The system was first equilibrated for 1 nanosecond (ns), followed by a 10 ns production simulation. During this period, sampling was performed every 500 fs to calculate ensemble averages. For visualization purposes, a snapshot of the system was taken every 1,000 fs. Using a threshold distance of 8 Å as the criterion for aggregation of adjacent molecular centers of mass, the number distribution of formed 4PACz and 4PA-Spiro clusters was calculated.[5]

The Buckingham potential is used to accurately capture the interactions of In_2O_3, which is a thoroughly validated classical force field developed by Walsh A et al.[6] The Consistent Valence Force Field (CVFF) in its all-atom form is employed to describe 4PACz and 4PA-Spiro. The van der Waals and electrostatic non-bonded interactions of 4PACz and 4PA-Spiro with In_2O_3 are calculated using real-space cutoff radii of 10 Å and 8 Å, respectively, while the particle-particle-particle-mesh (PPPM) method with an accuracy of 10^{-4}(kcal mol−1) $Å^{-1}$ is used to account for long-range electrostatic interactions. Geometric mixing rules are applied to all dissimilar non-bonded interactions.

Synthesis

Synthesis of 10H-spiro[acridine-9,9'-fluorene] (compound 1)

Diphenylamine (10 mmol, 1.69 g), p-Toluenesulfonic acid monohydrate (0.2 mmol, 38 mg) were added into a 100 mL flask. The reaction mixture was stirred for 15 min at 150 ^{o}C; Then, 9-Fluorenone (2.5 mmol, 0.45 g) was added into the mixture quickly. The reaction mixture was stirred for 30 min at 200 ^{o}C. After cooling to R.T., the reaction mixture was diluted with 40 mL of dichloromethane, then the solvent was removed under reduced pressure and the residue was purified by column chromatography (DCM/PE = 2:1, v/v) to afford white solid compound 1 (0.47 g, 57%). ^{1}H NMR (500 MHz, DMSO) δ 9.24 (s, 1H), 7.92 (d, J = 7.6 Hz, 2H), 7.37 (m, 2H), 7.29 – 7.14 (m, 4H), 7.05 (t, J = 7.6 Hz, 2H), 6.97 (d, J = 8.9 Hz, 2H), 6.55 – 6.43 (m, 2H), 6.11 (dd, J = 7.8, 0.9 Hz, 2H).

Synthesis of spiro-Br (compound 2)

10H-spiro[acridine-9,9'-fluorene] (compound 1, 1.2 mmol, 0.4 g), 1,4-dibromobutane (12 mmol, 0.26 g), tetrabuthylammonium bromide (0.2 mmol, 0.07 g) and 50% KOH aqueous solution (5 eq) were added into the flask. The reaction mixture was heated to 60 °C and kept stirring overnight under N_2. After cooling to R.T., the reaction mixture was extracted with dichloromethane. The organic layer was dried over anhydrous Na2SO4 and the solvent was distilled off under reduced pressure to give the crude

product. The crude product was simply purified by column chromatography (dichloromethane) to afford colorless oil (0.44 g, ~84%).

Notes:

- The 10H-spiro[acridine-9,9'-fluorene] is completely reacted, as confirmed by TLC. A little by-product that another bromine on spiro-Br is substituted by another spiro[acridine-9,9'-fluorene].
- Due to the by-product would not react and is easier separated in the next step, therefore, we collect a mixture in the step. Due to the potential participate the reaction in the next reaction, 1,4-dibromobutane is removed by column chromatography.

Synthesis of spiro-PP (compound 3)

spiro-Br (compound 2, ~0.94 mmol, 0.44 g) was dissolved in triethyl phosphite (20 eq, 18.8 mmol, 3.12 g) and the reaction mixture was heated at 140 ^{o}C and keep stirring overnight under N_2. After reaction completion, the solvent was distilled off under reduced pressure. The crude product was purified by column chromatography (dichloromethane and ethyl acetate successively) to give colorless oil (0.45 g, 91%).

Synthesis of 4PA-Spiro

spiro-PP (compound 3, 0.86 mmol, 0.45 g) was dissolved in anhydrous dichloromethane (10 mL) under N_2, then bromotrimethylsilane (10 eq) were added dropwise. The reaction mixture was stirred for overnight at R.T., then the solvent was distilled off under reduced pressure, the solid residue was reprecipitated. Afterwards, methanol (3 mL) was added and stirring for 3 h. Finally, distilled water was added dropwise (20 mL), until solution became opaque. Product was filtered off, dried and washed with dichloromethane to give white solid (0.35 g, 81%). ^{1}H NMR (400 MHz, DMSO-d_6) δ 7.90 (d, *J* = 7.6 Hz, 2H), 7.35 (td, *J* = 7.3, 1.6 Hz, 2H), 7.28 – 7.12 (m, 8H), 6.63 – 6.47 (m, 2H), 6.21 (dd, *J* = 7.7, 1.3 Hz, 2H), 4.20 – 3.95 (m, 2H), 1.94 (m, 2H), 1.87 – 1.59 (m, 4H). ^{13}C NMR (101 MHz, DMSO-d_6) δ 155.54, 140.47, 139.09, 128.83, 128.43, 128.24, 127.00, 126.43, 125.61, 120.78, 120.38, 113.66, 57.08, 45.61,

28.50, 27.14, 26.58, 26.43, 20.87. ^{31}P NMR (162 MHz, DMSO-d_6) δ 26.39. HRMS (ESI/QTOF) m/z: [M H-1]-Calcd for $C_{29}H_{25}NO_3P$-466.1578; Found 466.1573.

Notes: All the obtained intermediates were used for the next step, and the overall yield from initial raw material (10H-spiro[acridine-9,9'-fluorene]) is around 63%.

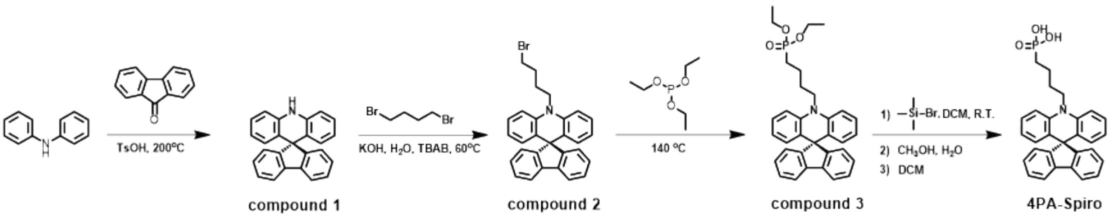

Scheme S1. Synthetic scheme of 4PA-Spiro.

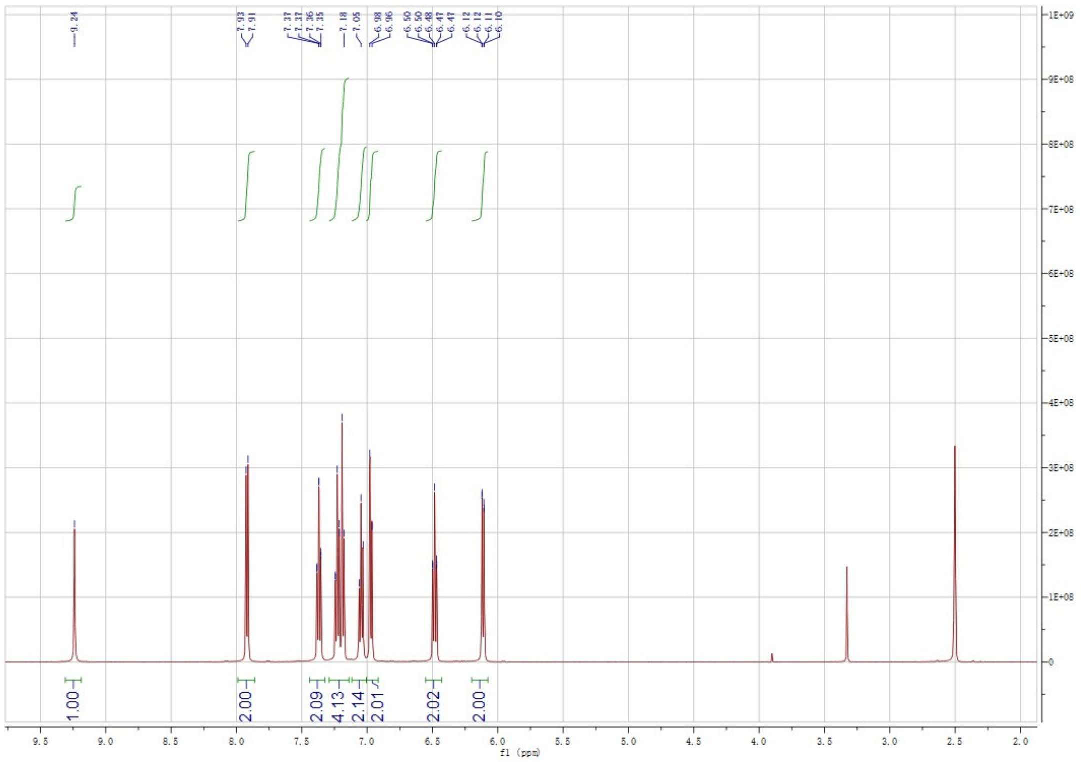

Figure S1. ^{1}H NMR spectrum of compound 1.

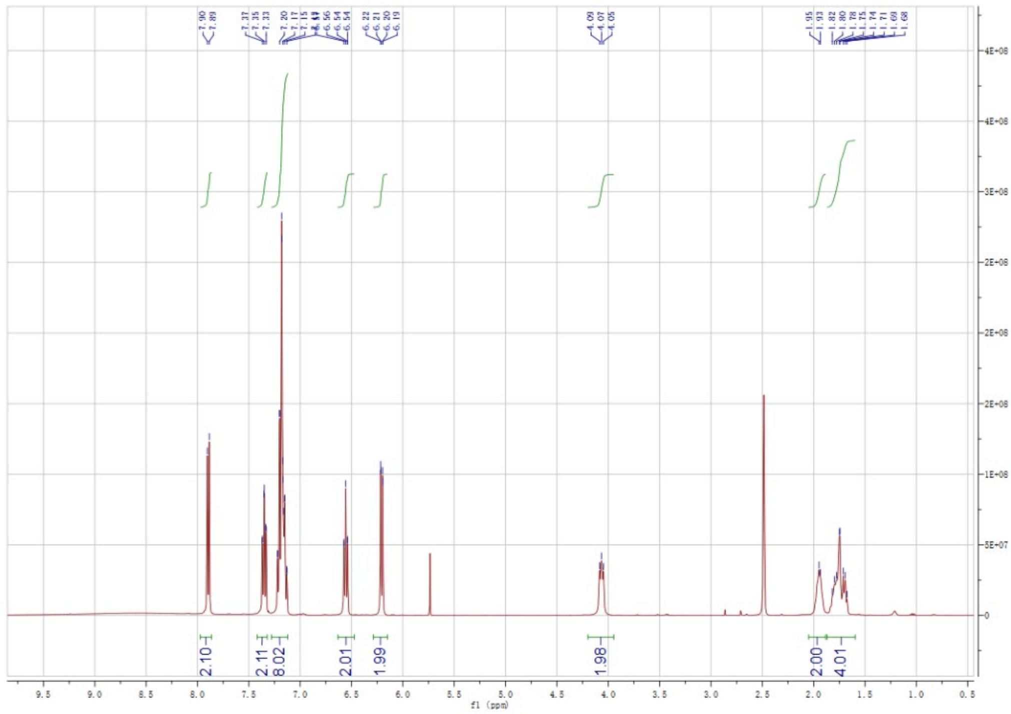

Figure S2. ^{1}H NMR spectrum of 4PA-Spiro.

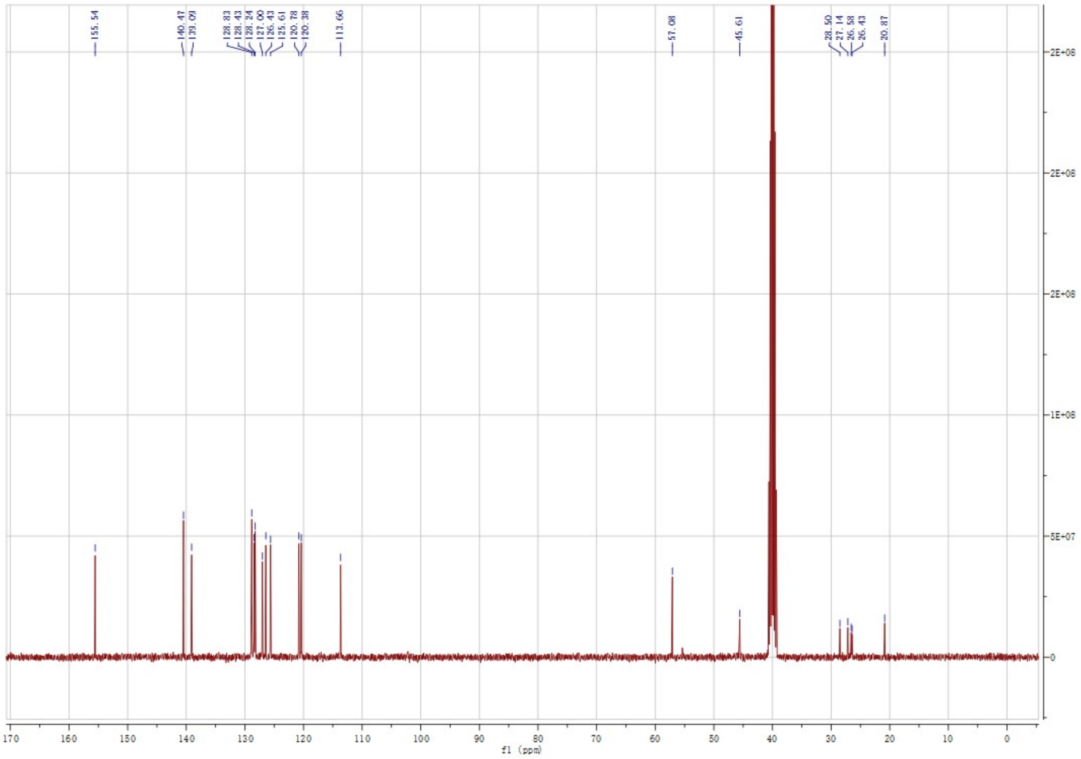

Figure S3. ^{13}C NMR spectrum of 4PA-Spiro.

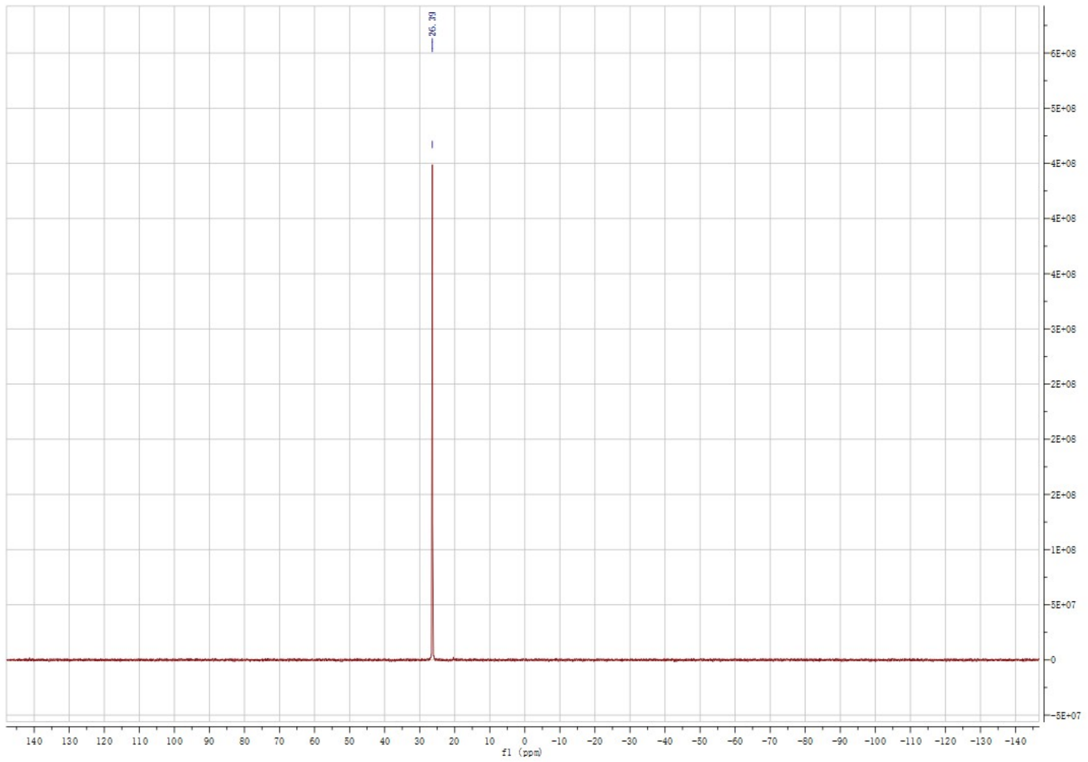

Figure S4. ^{31}P NMR spectrum of 4PA-Spiro.

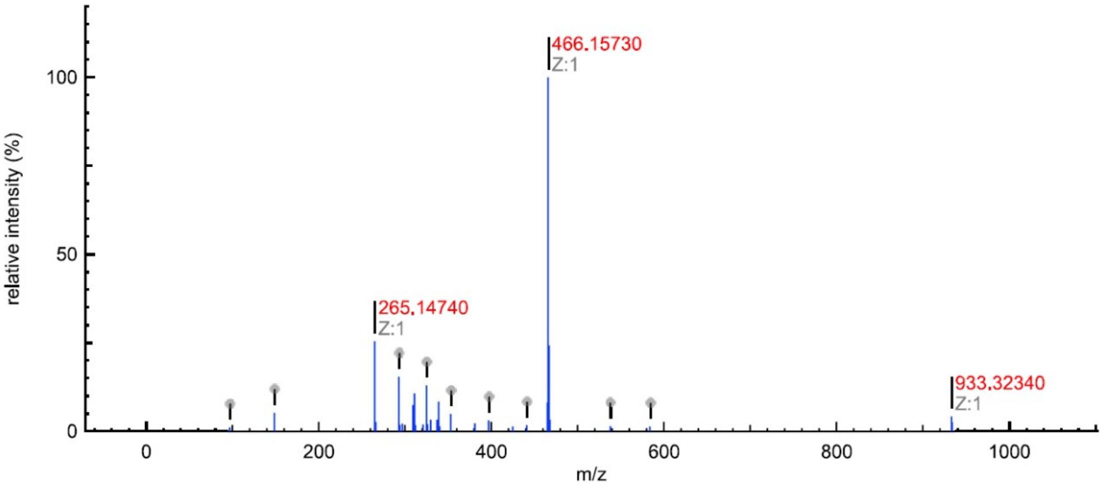

Figure S5. HRMS (ESI/QTOF) of 4PA-Spiro.

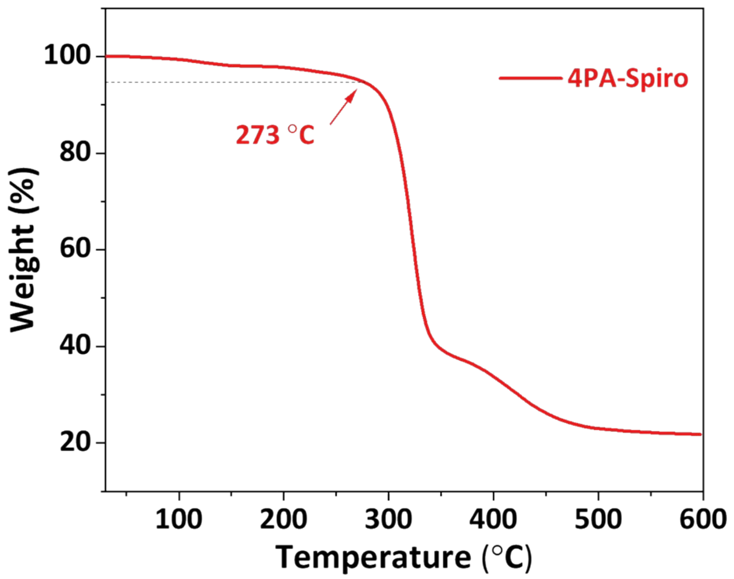

Figure S6. TGA of 4PA-Spiro.

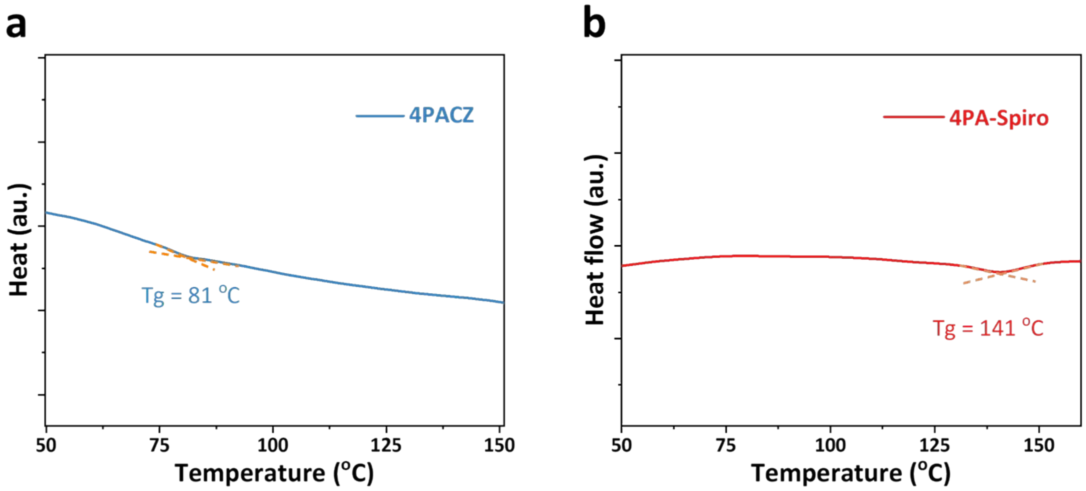

Figure S7. Differential scanning calorimetry of (a) 4PACz and (b) 4PA-Spiro.

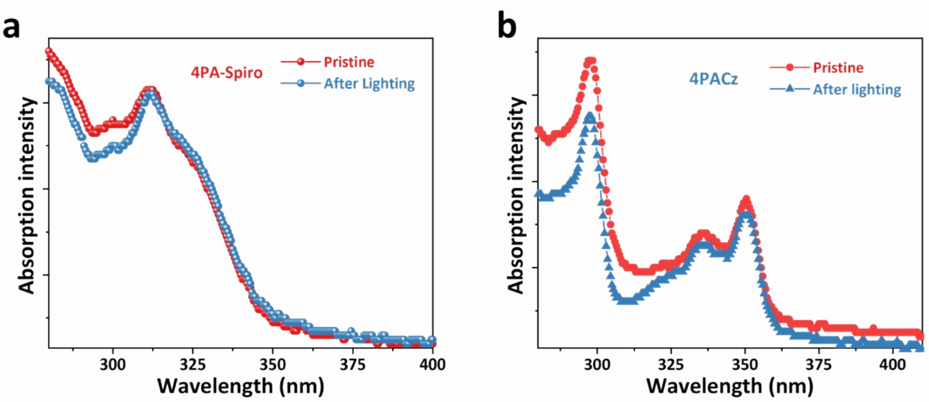

Figure S8. UV-vis absorption spectra of (a) 4PA-Spiro and (b) 4PACz films before and after one-sun irradiation for 100 hours.

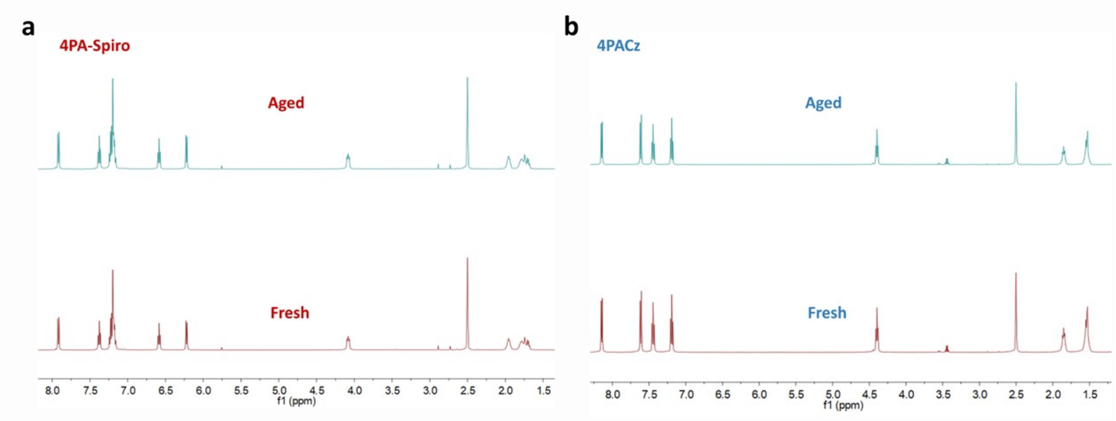

Figure S9. ^1H NMR spectra of (a) 4PA-Spiro and (b) 4PACz before and after one-sun irradiation for 100 hours.

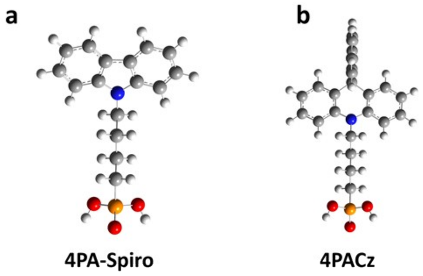

Figure S10. Optimized structure of (a) 4PA-Spiro and 4PACz from top view.

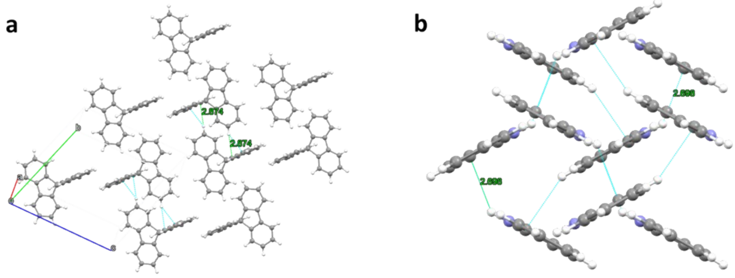

Figure S11. Single-crystal of (a) spirobifluorene and (b) 9H-carbzole.

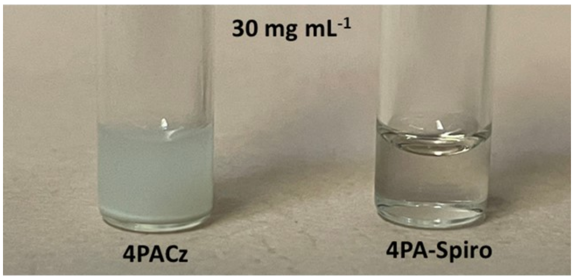

Figure S12. Solubility of 4PACz and 4PA-Spiro dissolved in DMF.

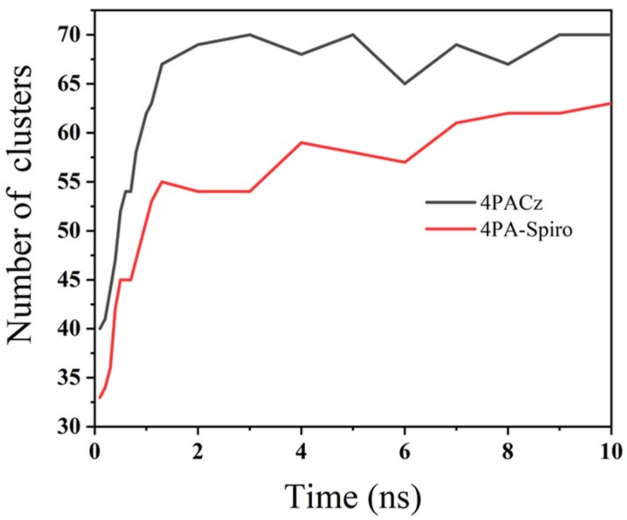

Figure S13. Total number of 4PACz and 4PA-Spiro clusters formed over a set period.

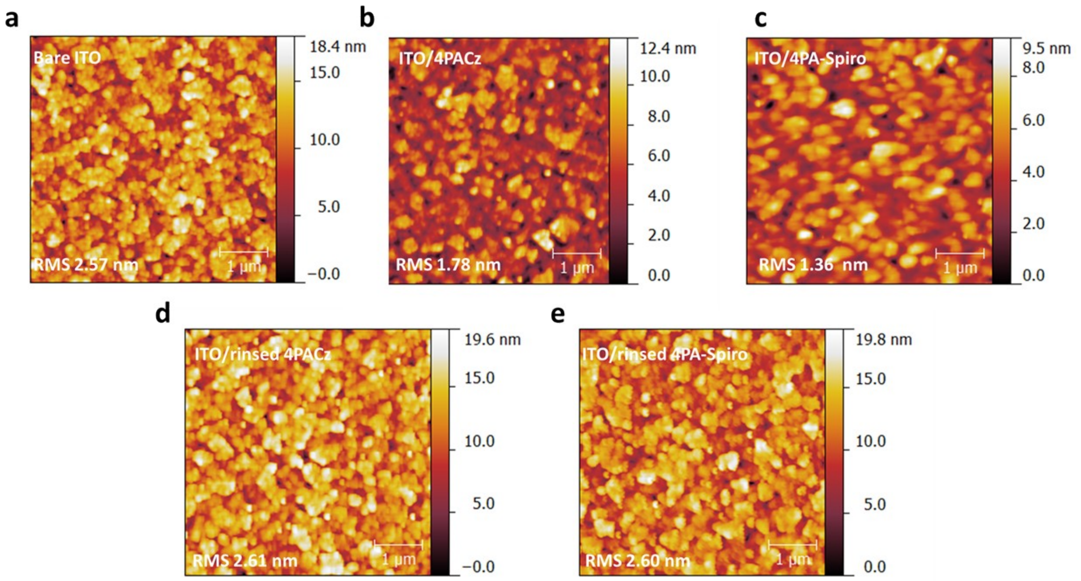

Figure S14. AFM measurement of the (a) bare ITO, (b) 4PACZ, (c) 4PA-Spiro film, and the (d) rinsed 4PACZ and (e) rinsed 4PA-Spiro with DMF solvent.

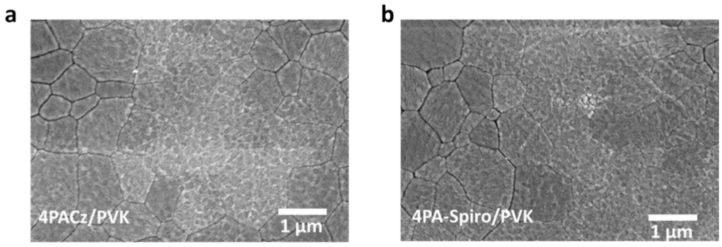

Figure S15. SEM images of the perovskite buried interface coated on 4PACz and 4PA-Spiro.

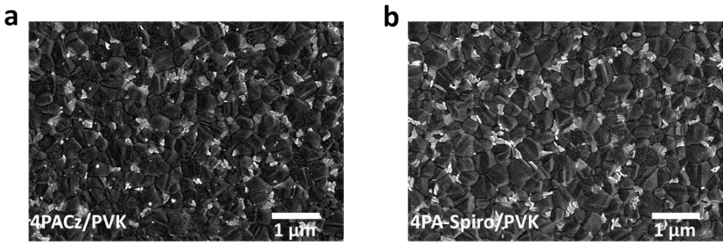

Figure S16. SEM images of top surface of the perovskite (PVK) films on the (a) 4PACz and (b) 4PA-Spiro coated ITO substrate.

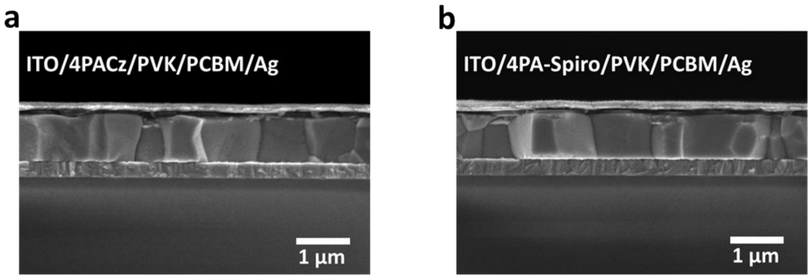

Figure S17. Cross-sectional SEM images of PSCs with (a) 4PACz and (b) 4PA-Spiro as HTMs.

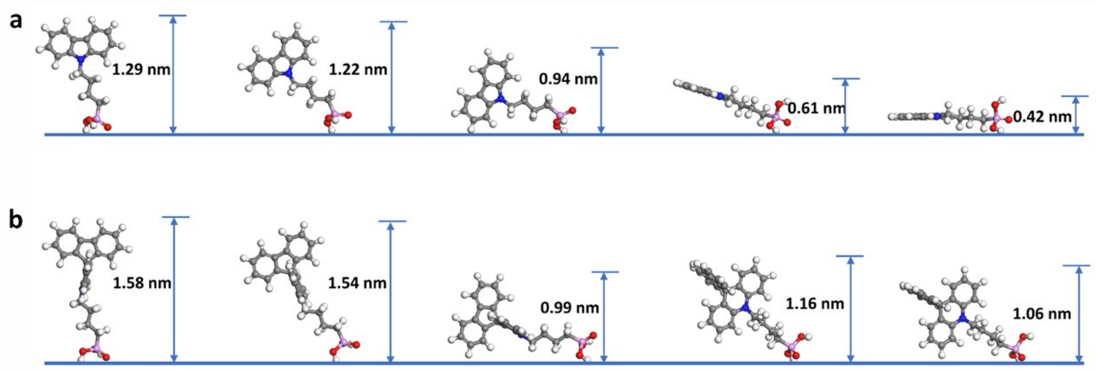

Figure S18. The heights of different stacking arrangements of (a) 4PACz and 4PA-Spiro on the substrate.

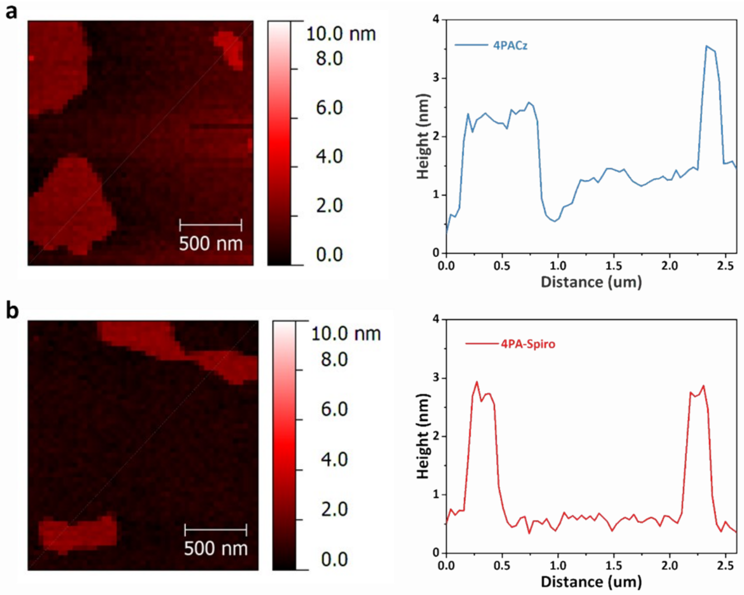

Figure S19. Atomic force microscopy (AFM) measurements of 4PACz and 4PA-Spiro deposited on the silicon wafer substrate.

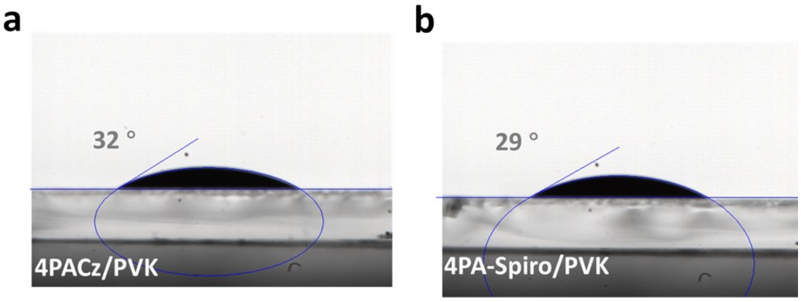

Figure S20. Perovskite precursor solution contact angles of 4PACz and 4PA-Spiro coated on the ITO substrate.

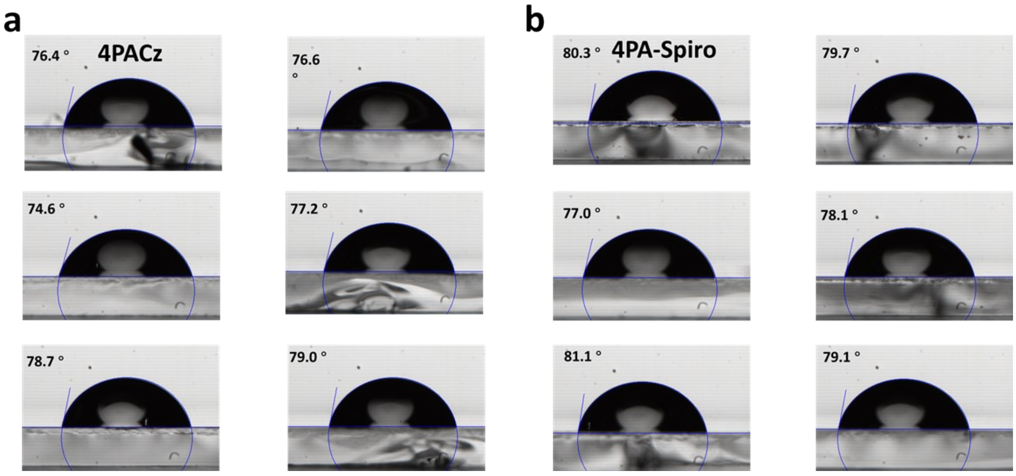

Figure S21. Water contact angles of 4PACz and 4PA-Spiro coated ITO substrate.

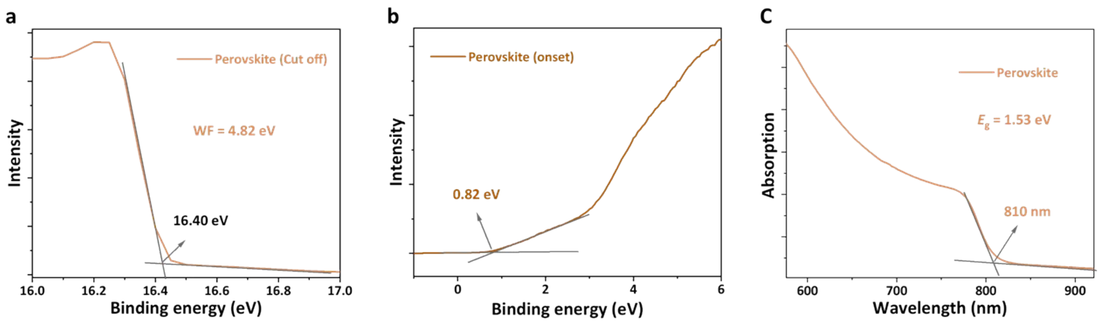

Figure S22. (a) Cut-off and (c) onset region of UPS spectra for perovskite. (c) UV-vis absorption spectra of the perovskite film.

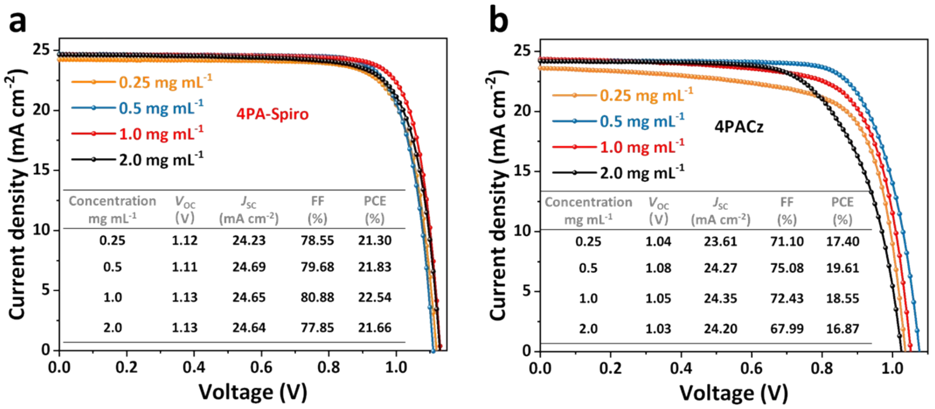

Figure S23. *J-V* curves of the devices with various concentrations of (a) 4PA-Cz, (b) 4PACz in DMF.

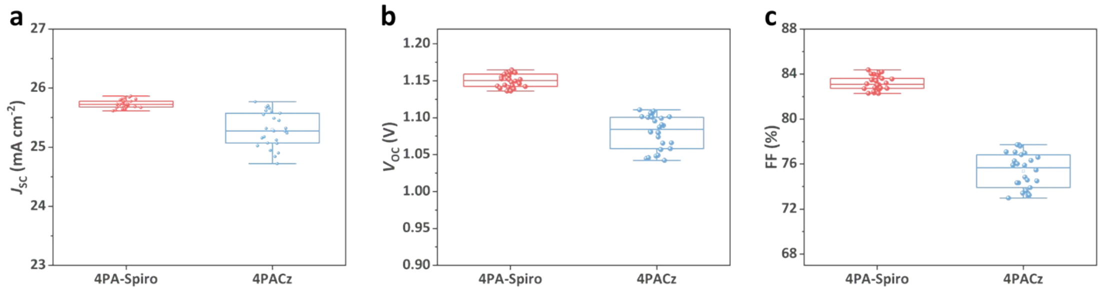

Figure S24. Statistical distribution of J_{SC}, V_{OC} and FF for PSCs (0.05 cm^2) with different SAMs.

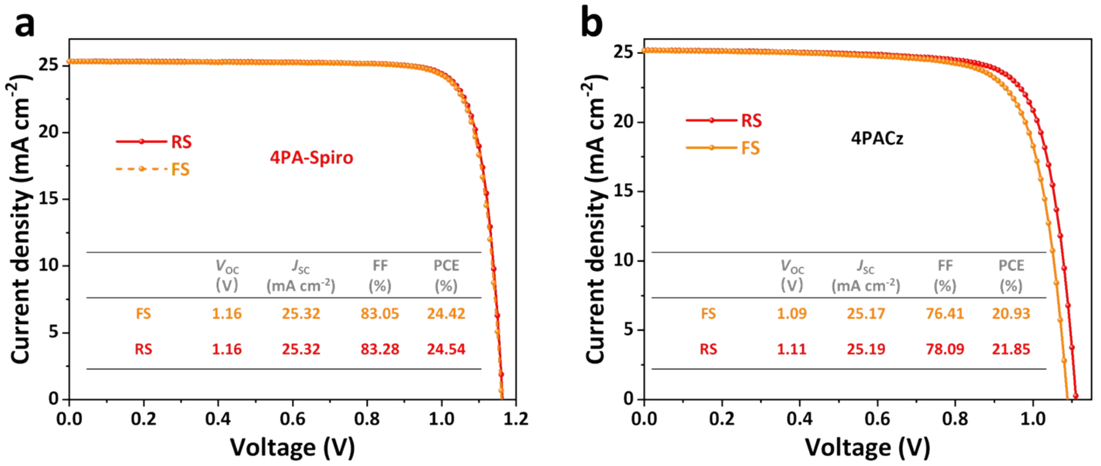

Figure S25. Hysteresis behavior of the (a) 4PA-Spiro- and (b) 4PACz-based devices with different scan direction.

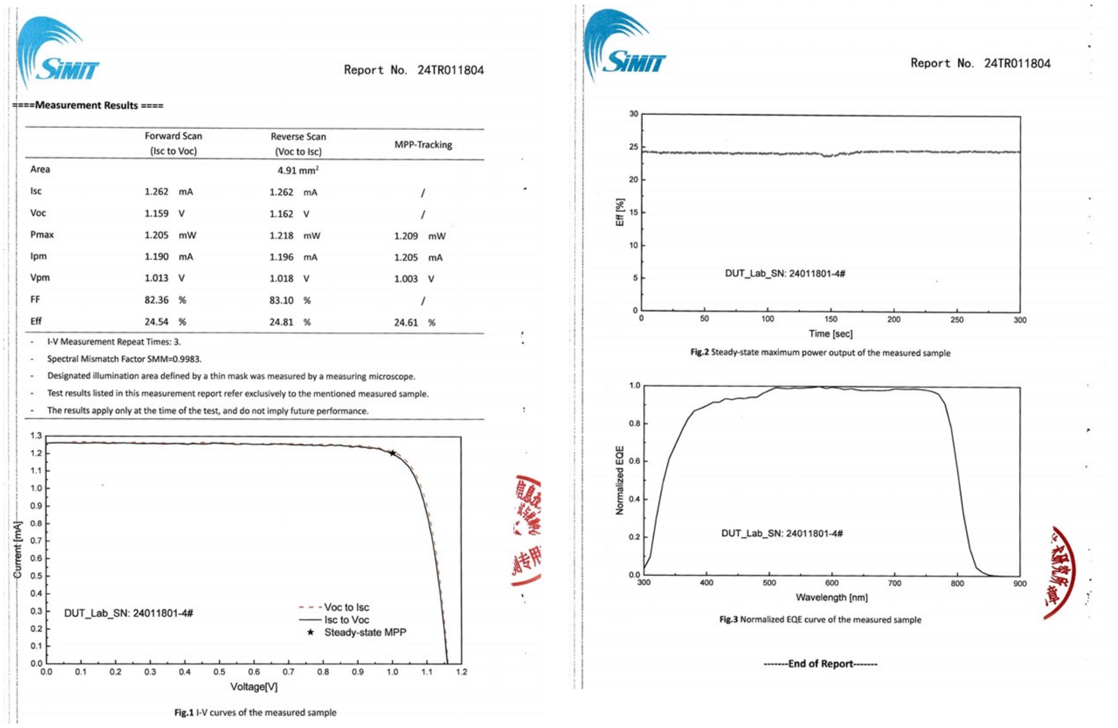
Report No. 24TR011804

====Measurement Results ====

	Forward Scan (Isc to Voc)	Reverse Scan (Voc to Isc)	MPP-Tracking
Area		4.91 mm²	
Isc	1.262 mA	1.262 mA	/
Voc	1.159 V	1.162 V	/
Pmax	1.205 mW	1.218 mW	1.209 mW
Ipm	1.190 mA	1.196 mA	1.205 mA
Vpm	1.013 V	1.018 V	1.003 V
FF	82.36 %	83.10 %	/
Eff	24.54 %	24.81 %	24.61 %

- I-V Measurement Repeat Times: 3.
- Spectral Mismatch Factor SMM=0.9983.
- Designated illumination area defined by a thin mask was measured by a measuring microscope.
- Test results listed in this measurement report refer exclusively to the mentioned measured sample.
- The results apply only at the time of the test, and do not imply future performance.

Fig.1 I-V curves of the measured sample

Report No. 24TR011804

Fig.2 Steady-state maximum power output of the measured sample

Fig.3 Normalized EQE curve of the measured sample

-------End of Report-------

Figure S26. Certified performance of PSCs based on SAM of 4PA-Spiro from Shanghai Institute of Microsystem and Information Technology (*SIMIT*), China.

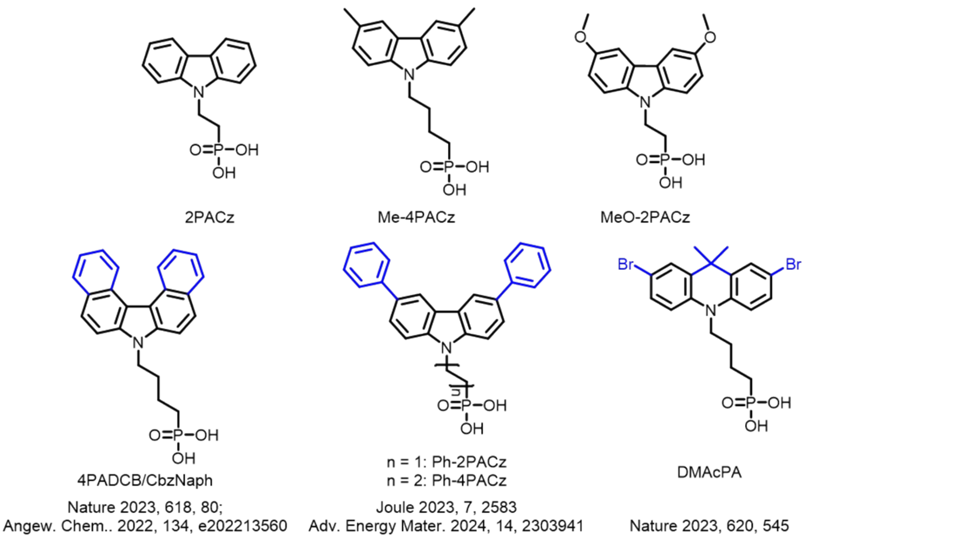

Figure S27. Molecular structures of typically reported SAMs used for highly performance PSCs.

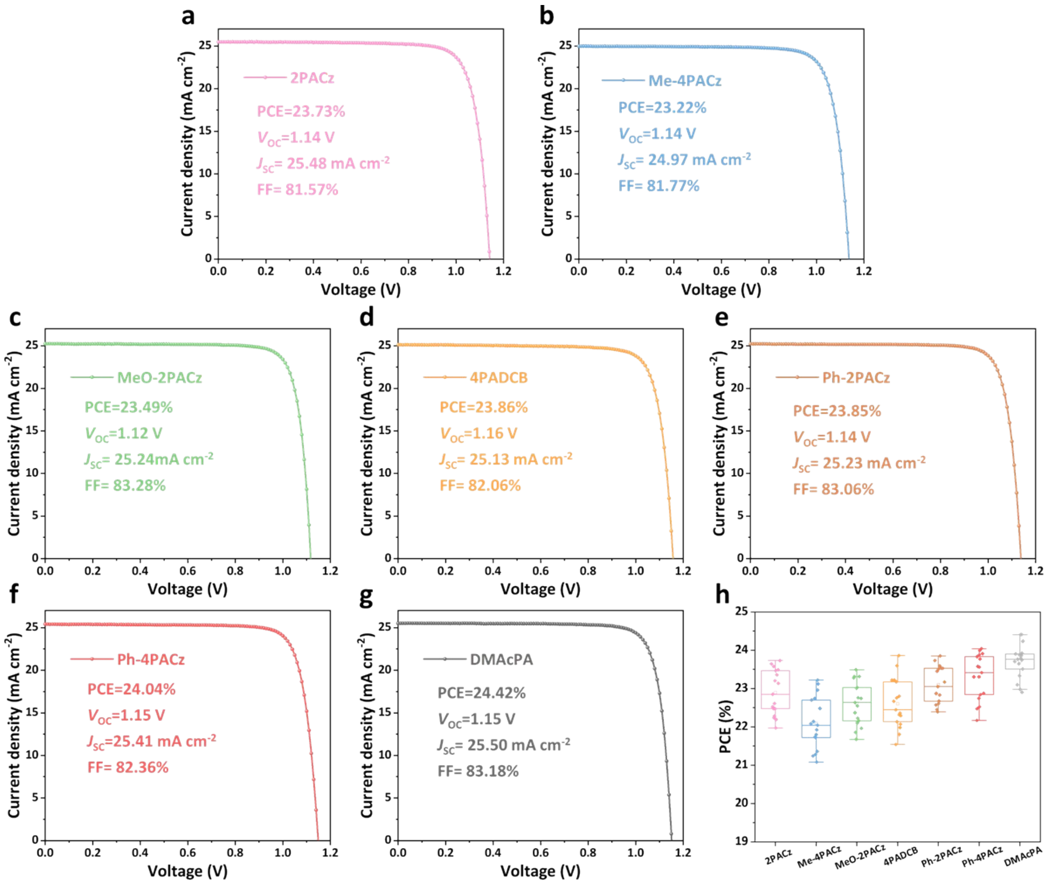

Figure S28. *J-V* curves of PSCs with (a) 2PACZ, (b) Me-4PACz, (c) MeO-2PACZ, (d) 4PADCB, (e) Ph-2PACZ, (f)Ph-4PACz and (g) DMAcPA. (h) statistic of PCEs for PSCs with different SAMs.

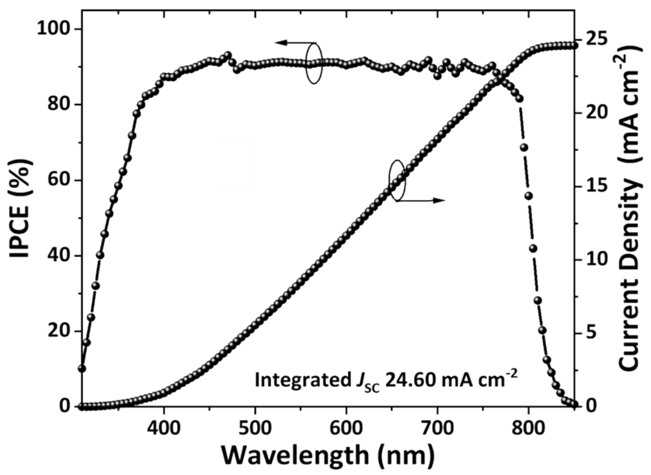

Figure S29. IPCE and integrated current density of the 4PACz-based device.

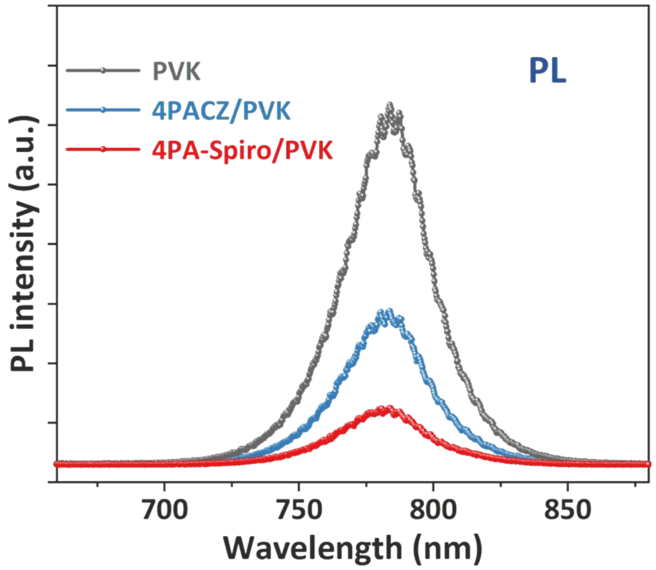

Figure S30. Steady-state PL spectra of perovskite on SAMs at the buried interface.

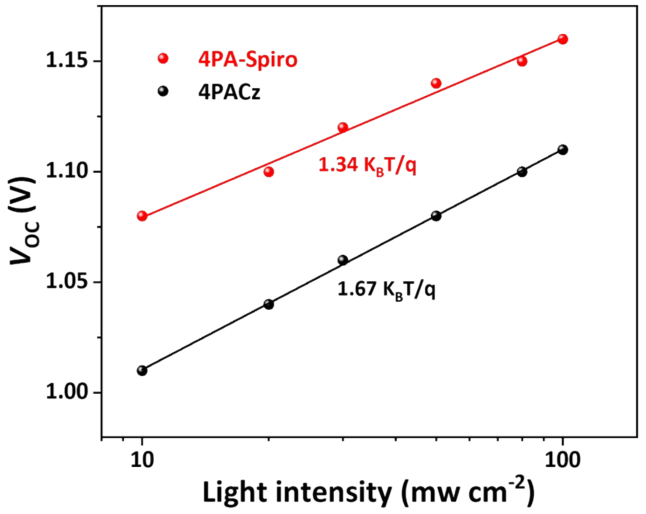

Figure S31. Light intensity dependent V_{OC} of the 4PACz- and 4PA-Spiro-based devices.

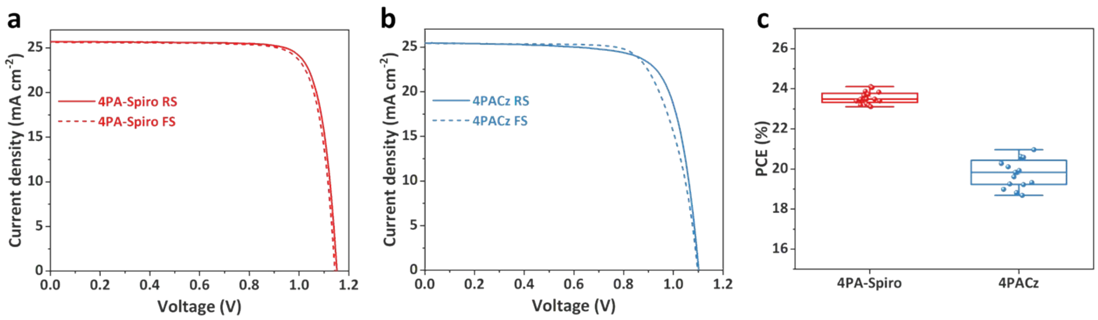

Figure S32. Hysteresis behavior of the (a) 4PA-Spiro- and (b) 4PACz-based devices (1.0 cm^2) with different scan direction. (c) statistic of PCEs for PSCs (1.0 cm^2) with different SAMs.

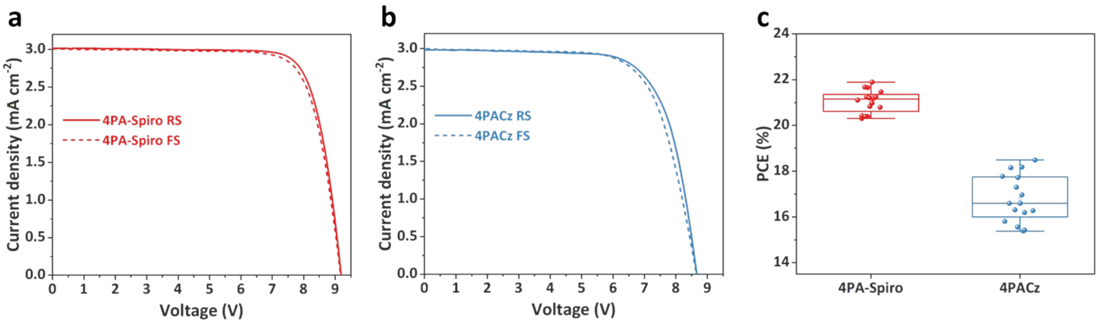

Figure S33. Hysteresis behavior of the (a) 4PA-Spiro- and (b) 4PACz-based modules (29.0 cm^2) with different scan direction. (c) statistic of PCEs for PSCs modules (29.0 cm^2) with different SAMs.

A simple analysis of relative costs of 4PA-Spiro

The lab synthesis cost of 4PA-Spiro are estimated on a model originally proposed by Osedach *et al.*[7] Recently, Pertrus and Malinauskas *et al.*[8, 9] has used the model to estimate the cost of hole transporting materials. For every synthetic step the required amounts of reactants, catalysts, reagents and solvents are calculated to obtain 1 gram of 4PA-Spiro are reported (Table S1-4).

Table S1. Synthesis cost of compound 1.

Chemical	Weight Reagent or solvent (g/g)	Price of chemical ($/g)	Cost of chemical ($/g product)	Total cost ($/g)
Diphenylamine	3.60	0.03	0.1	
p-Toluenesulfonic acid monohydrate	0.08	0.15	0.01	
9-Fluorenone	0.96	0.03	0.03	
petroleum ether	200	0.0028	0.56	
dichloromethane	400	0.0028	1.12	
				~1.82

Table S2. Synthesis cost of compound 2.

Chemical	Weight Reagent or solvent (g/g)	Price of chemical ($/g)	Cost of chemical ($/g product)	Total cost ($/g)
10H-spiro[acridine-9,9'-fluorene]	0.91	1.82	1.66	
1,4-dibromobutane	0.59	0.08	0.05	
tetrabuthylammonium bromide	0.16	0.03	0.01	
KOH	0.2	0.01	<0.01	
Na2SO4	0.4	<0.01	<0.01	
dichloromethane	300	0.0028	0.84	
				~2.58

Table S3. Synthesis cost of compound 3.

Chemical	Weight Reagent or solvent (mL or g/g)	Price of chemical ($/g or mL, source)	Cost of chemical ($/g product)	Total cost ($/g)
compound 2	0.98	2.58	2.53	
triethyl phosphite	6.93	0.07	0.49	
dichloromethane	200	0.0028	0.56	
ethyl acetate	200	0.005	1.00	
				~4.58

Table S4. Synthesis cost of 4PA-Spiro.

Chemical	Weight Reagent or solvent (mL or g/g)	Price of chemical ($/g or mL, source)	Cost of chemical ($/g product)	Total cost ($/g)
compound 3	1.29	4.58	5.91	
dichloromethane	40	0.0028	0.11	
bromotrimethylsilane	5	0.1	0.5	
methanol	5	0.0028	0.01	
				~6.53

Table S5. A brief summary of inverted PSC modules.

HTMs	Devices Structure	PCE (%)	Area (cm^2)	Year	Ref
NiOx	FTO/Urea-NiOx /perovskite /C60/BCP/Cu	18.97	16	2023	[10]
NiOx/PTAA -0.2P	ITO/HTL /perovskite /C60/BCP/Ag	20.7	19.4	2023	[11]
NiOx/Me-4PACz	FTO/NiOx/Me-4PACz/FAPbI3/PCBM/SnO2/Cu	21	14.6	2023	[12]
MPA-CPA	ITO/HTL/perovskite/C60/BCP/Ag	22.0	10	2023	[13]
PTAA	ITO/HTL/perovskite/C60/BCP/Cu	21.8	26.9	2023	[14]
NiOx	ITO/NiOx/$MAPbI_3$/PCBM/BCP/Cu	19.03	18.1	2023	[15]
NiOx	ITO/NiOx/perovskite/PC61BM/BCP/Ag	20.58	63.74	2024	[16]
PTAA	ITO/HTL/perovskite/C60/BCP/Cu	20.23	108	2024	[17]
**4PA-Spiro**	**ITO/4PA-Spiro/perovskite/PCBM/BCP/Ag**	**21.87**	**29.0**	**This work**	

References

1. Q. Tan, Z. Li, G. Luo, X. Zhang, B. Che, G. Chen, H. Gao, D. He, G. Ma, J. Wang, J. Xiu, H. Yi, T. Chen and Z. He, *Nature*, 2023, **620**, 545-551.
2. Y. Ding, B. Ding, H. Kanda, O. J. Usiobo, T. Gallet, Z. Yang, Y. Liu, H. Huang, J. Sheng, C. Liu, Y. Yang, V. I. E. Queloz, X. Zhang, J.-N. Audinot, A. Redinger, W. Dang, E. Mosconic, W. Luo, F. De Angelis, M. Wang, P. Dörflinger, M. Armer, V. Schmid, R. Wang, K. G. Brooks, J. Wu, V. Dyakonov, G. Yang, S. Dai, P. J. Dyson and M. K. Nazeeruddin, *Nat. Nanotechnol.*, 2022, **17**, 598-605.
3. A. P. Thompson, H. M. Aktulga, R. Berger, D. S. Bolintineanu, W. M. Brown, P. S. Crozier, P. J. in 't Veld, A. Kohlmeyer, S. G. Moore, T. D. Nguyen, R. Shan, M. J. Stevens, J. Tranchida, C. Trott and S. J. Plimpton, *Comput. Phys. Commun.*, 2022, **271**, 108171.
4. W. C. Swope, H. C. Andersen, P. H. Berens and K. R. Wilson, *J. Chem. Phys.*, 1982, **76**, 637-649.
5. S. M. Park, M. Wei, N. Lempesis, W. Yu, T. Hossain, L. Agosta, V. Carnevali, H. R. Atapattu, P. Serles, F. T. Eickemeyer, H. Shin, M. Vafaie, D. Choi, K. Darabi, E. D. Jung, Y. Yang, D. B. Kim, S. M. Zakeeruddin, B. Chen, A. Amassian, T. Filleter, M. G. Kanatzidis, K. R. Graham, L. Xiao, U. Rothlisberger, M. Grätzel and E. H. Sargent, *Nature*, 2023, **624**, 289-294.
6. A. Walsh, C. R. A. Catlow, A. A. Sokol and S. M. Woodley, *Chem. Mater.*, 2009, **21**, 4962-4969.
7. T. P. Osedach, T. L. Andrew and V. Bulović, *Energy Environ. Sci.*, 2013, **6**, 711-718.
8. M. L. Petrus, T. Bein, T. J. Dingemans and P. Docampo, *J. Mater. Chem. A*, 2015, **3**, 12159-12162.
9. T. Malinauskas, M. Saliba, T. Matsui, M. Daskeviciene, S. Urnikaite, P. Gratia, R. Send, H. Wonneberger, I. Bruder, M. Graetzel, V. Getautis and M. K. Nazeeruddin, *Energy Environ. Sci.*, 2016, **9**, 1681-1686.
10. C. Li, Y. Zhang, X. Zhang, P. Zhang, X. Yang and H. Chen, *Adv. Funct. Mater.*,

2023, **33**, 2214774.

11. B. Niu, H. Liu, Y. Huang, E. Gu, M. Yan, Z. Shen, K. Yan, B. Yan, J. Yao, Y. Fang, H. Chen and C.-Z. Li, *Adv. Mater.*, 2023, **35**, 2212258.
12. S. Yu, Z. Xiong, H. Zhou, Q. Zhang, Z. Wang, F. Ma, Z. Qu, Y. Zhao, X. Chu, X. Zhang and J. You, *Science*, 2023, **382**, 1399-1404.
13. S. Zhang, F. Ye, X. Wang, R. Chen, H. Zhang, L. Zhan, X. Jiang, Y. Li, X. Ji, S. Liu, M. Yu, F. Yu, Y. Zhang, R. Wu, Z. Liu, Z. Ning, D. Neher, L. Han, Y. Lin, H. Tian, W. Chen, M. Stolterfoht, L. Zhang, W.-H. Zhu and Y. Wu, *Science*, 2023, **380**, 404-409.
14. C. Fei, N. Li, M. Wang, X. Wang, H. Gu, B. Chen, Z. Zhang, Z. Ni, H. Jiao, W. Xu, Z. Shi, Y. Yan and J. Huang, *Science*, 2023, **380**, 823-829.
15. S. Li, X. Wang, H. Li, J. Fang, D. Wang, G. Xie, D. Lin, S. He and L. Qiu, *Small*, 2023, **19**, 2301110.
16. Z. Zhang, M. Li, R. Li, X. Zhuang, C. Wang, X. Shang, D. He, J. Chen and C. Chen, *Adv. Mater.*, 2024, **36**, 2313860..
17. M. A. Uddin, P. J. S. Rana, Z. Ni, G. Yang, M. Li, M. Wang, H. Gu, H. Zhang, B. D. Dou and J. Huang, *Nat. Commun.*, 2024, **15**, 1355.
